# Medicare eligibility and healthcare access, affordability, and financial strain for low- and higher-income adults in the United States: A regression discontinuity analysis

**DOI:** 10.1371/journal.pmed.1004083

**Published:** 2022-10-04

**Authors:** Rahul Aggarwal, Robert W. Yeh, Issa J. Dahabreh, Sarah E. Robertson, Rishi K. Wadhera

**Affiliations:** 1 Richard A. and Susan F. Smith Center for Outcomes Research, Beth Israel Deaconess Medical Center and Harvard Medical School, Boston, Massachusetts, United States of America; 2 Division of Cardiovascular Medicine, Brigham and Women’s Hospital and Harvard Medical School, Boston, Massachusetts, United States of America; 3 CAUSALab, Harvard T.H. Chan School of Public Health, Boston, Massachusetts, United States of America; 4 Department of Epidemiology, Harvard T.H. Chan School of Public Health, Boston, Massachusetts, United States of America; 5 Department of Biostatistics, Harvard T.H. Chan School of Public Health, Boston, Massachusetts, United States of America; Washington University in St Louis School of Medicine, UNITED STATES

## Abstract

**Background:**

US policymakers are debating whether to expand the Medicare program by lowering the age of eligibility. The goal of this study was to determine the association of Medicare eligibility and enrollment with healthcare access, affordability, and financial strain from medical bills in a contemporary population of low- and higher-income adults in the US.

**Methods and findings:**

We used cross-sectional data from the National Health Interview Survey (2019) to examine the association of Medicare eligibility and enrollment with outcomes by income status using a local randomization-based regression discontinuity approach. After weighting to account for survey sampling, the low-income group consisted of 1,660,188 adults age 64 years and 1,488,875 adults age 66 years, with similar baseline characteristics, including distribution of sex (59.2% versus 59.7% female) and education (10.8% versus 12.5% with bachelor’s degree or higher). The higher-income group consisted of 2,110,995 adults age 64 years and 2,167,676 adults age 66 years, with similar distribution of baseline characteristics, including sex (40.0% versus 49.4% female) and education (41.0% versus 41.6%). The share of adults age 64 versus 66 years enrolled in Medicare differed within low-income (27.6% versus 87.8%, *p* < 0.001) and higher-income groups (8.0% versus 85.9%, *p* < 0.001). Medicare eligibility at 65 years was associated with a decreases in the percentage of low-income adults who delayed (14.7% to 6.2%; −8.5% [95% CI, −14.7%, −2.4%], *P* = 0.007) or avoided medical care (15.5% to 5.9%; −9.6% [−15.9%, −3.2%], *P* = 0.003) due to costs, and a larger decrease in the percentage who were worried about (66.5% to 51.1%; −15.4% [−25.4%, −5.4%], *P* = 0.003) or had problems (33.9% to 20.6%; −13.3% [−23.0%, −3.6%], *P* = 0.007) paying medical bills. In contrast, there were no significant associations between Medicare eligibility and measures of cost-related barriers to medication use. For higher-income adults, there was a large decrease in worrying about paying medical bills (40.5% to 27.5%; −13.0% [−21.4%, −4.5%], *P* = 0.003), a more modest decrease in avoiding medical care due to cost (3.5% to 0.6%; −2.9% [−5.3%, −0.5%], *P* = 0.02), and no significant association between eligibility and other measures of healthcare access and affordability. All estimates were stronger when examining the association of Medicare enrollment with outcomes for low and higher-income adults. Additional analyses that adjusted for clinical comorbidities and employment status were largely consistent with the main findings, as were analyses stratified by levels of educational attainment. Study limitations include the assumption adults age 64 and 66 would have similar outcomes if both groups were eligible for Medicare or if eligibility were withheld from both.

**Conclusions:**

Medicare eligibility and enrollment at age 65 years were associated with improvements in healthcare access, affordability, and financial strain in low-income adults and, to a lesser extent, in higher-income adults. Our findings provide evidence that lowering the age of eligibility for Medicare may improve health inequities in the US.

## Abstract 2

Rahul Aggarwal and colleagues explore the association of Medicare eligibility and enrollment with health care access, affordability, and financial strain from medical bills in low- and higher-income adults in the US.

## Author summary

**Why was this study done?:**

- In the United States, low-income adults are more likely to lack health insurance coverage, face barriers accessing healthcare, and disproportionately experience financial strain due to healthcare expenditures compared with higher-income adults.
- Policymakers are increasingly debating whether to expand the Medicare program by lowering the age of eligibility.
- We determine if Medicare eligibility and enrollment at age 65 years is associated with changes in healthcare access, affordability, and financial strain for low-income and higher-income adults.

**What did the researchers do and find?:**

- In this cross-sectional study, Medicare eligibility and enrollment were associated with decreases in the percentage of low-income adults who delayed or avoided medical care due to costs, as well as in the percentage whom were worried about or had problems paying medical bills.
- These findings were more modest and less consistent in higher-income adults.
- Medicare was not associated with changes in the percentage of low-income or higher-income adults who experienced cost-related barriers to medication use.

**What do these findings mean?:**

- As the debate on lowering the age of Medicare eligibility continues, policymakers should consider the potential implications of doing so on healthcare access and affordability for low-income adults and, more broadly, health equity in the US.

## Introduction

The Medicare program provides health insurance coverage to more than 50 million older adults in the United States [1]. Medicare enjoys broad public support and is viewed favorably among the majority of beneficiaries [2]. As a result, policymakers have increasingly called for the expansion of the Medicare program, including recent proposals to lower the age of eligibility from 65 to 60 years [3,4].

One group that may significantly benefit from reducing the age of Medicare eligibility are low-income adults, who have worse health outcomes and higher rates of morbidity than higher-income adults in the US [5–7]. Low-income adults are more likely to be uninsured, face challenges in healthcare access, and disproportionately experience financial strain due to healthcare expenditures [8]. Although Medicaid coverage was expanded under the Affordable Care Act (ACA) for those making less than 138% of the federal poverty level (FPL), many low-income adults remain uninsured because they do not meet eligibility criteria or reside in non-expansion states. At the same time, more than 40% of working-age adults are now inadequately insured (underinsurance) [9], and many Americans face rising out-of-pocket costs, due in part to higher premiums, the rapid growth of high-deductible health plans, and changes in benefit design over the last decade.[10] While prior studies suggest that Medicare eligibility leads to changes in healthcare access and utilization [11,12], little is known about the effects of Medicare on low- and higher-income adults in the ACA era, which is particularly important given the changes that have occurred in the healthcare landscape over the past decade (rising “underinsurance,” expansion of the Medicaid program) and rising income inequality in the US.

As policymakers continue to debate whether the Medicare program should be expanded [3], it will be critical to understand whether Medicare is associated with healthcare access, affordability, and financial strain in a contemporary population of low-income adults in the US, and if this association differs for higher-income adults. Therefore, in this study, we examined the association of Medicare eligibility and enrollment at age 65 years with healthcare access, prescription medication use, and financial strain due to medical bills in low-income adults, as well as their higher-income counterparts, using a local randomization-based regression discontinuity approach. In addition, we also evaluated the association of Medicare with outcomes for adults with low and higher levels of educational attainment.

## Methods

### Study population

We used data from the National Health Interview Survey (NHIS), a nationally representative health surveillance survey conducted by the US Census Bureau on behalf of the National Center for Health Statistics [13]. We only included data from the 2019 cycle of NHIS to obtain estimates reflective of a contemporary population. The survey uses a geographically clustered sampling technique to select a random sample of households from 50 US states and the District of Columbia. Adults (≥18 years) are randomly selected from households for a detailed interview (*N* = 31,997) [14]. Our study population included US adults age 64 years, the majority of whom are not eligible for Medicare, and those age 66 years, who are eligible for Medicare. Because age is provided in discrete years by NHIS, we removed from the analysis individuals who appear in the data as being age 65 years, because their responses could reflect outcomes at age 64 (prior to eligibility). We defined low-income adults as those residing in households with an income of ≤300% of the FPL and higher-income adults as those in households with an income of >300% of the FPL, consistent with prior reports [15,16] (we also used an alternative definition of ≤200% FPL to define low-income as a sensitivity analysis; described below). In addition, we also identified adults with low versus higher levels of educational attainment (high school or less versus Bachelor’s degree or higher, respectively).

### Study design and identification strategy

To examine the association of Medicare eligibility or enrollment with outcomes for each income group, we took advantage of changes in the criteria for Medicare eligibility at age 65. The majority of individuals in the US become eligible for Medicare coverage at age 65 years or older. This policy induces a discontinuity in the probability of enrolling in Medicare around the cutoff of age of 65 years: individuals younger than age 65 are much less likely to be enrolled in the program compared to individuals older than age 65. At the same, time individuals on either side of the cutoff, but close to it (e.g., age 64 years versus 66 years), are likely to be similar except for Medicare eligibility.

The similarity of the 2 groups may support a “local randomization assumption” [17,18]: Individuals close to the cutoff are approximately exchangeable with respect to their potential outcomes (counterfactuals) under interventions to change eligibility or enrollment [19,20]. More specifically, we can assume that (1) individuals just above the cutoff would experience outcomes similar to individuals just below the cutoff, under a hypothetical intervention to expand coverage to both and under a hypothetical intervention to withhold coverage from both; and (2) individuals just above the cutoff would have obtained coverage similar to individuals just below the cutoff, under a hypothetical intervention to expand eligibility to both and under a hypothetical intervention to withhold eligibility from both. Comparisons of baseline characteristics of the 2 groups can be used to assess the plausibility of the local randomization assumption. We only included narrow age intervals above and below the threshold to minimize potential violations of the local randomization assumption (i.e., adults 60 years of age are less similar to adults 66 years of age than adults 64 years of age).

Under the local randomization assumption, we compared outcomes among individuals just above (age 66 years) and below (age 64 years) the cutoff (i.e., Medicare eligible and non-eligible individuals), thus attempting to approximate the results of a target trial that would randomize individuals with age close to the cutoff to being offered versus not being offered Medicare enrollment (i.e., being eligible versus not). In addition, we used the discontinuity in the probability of Medicare enrollment around the eligibility cutoff to estimate the effect of Medicare enrollment (see Statistical analysis for details).

### Outcome measures

We examined outcomes across 3 domains: healthcare access, prescription medication use, and financial strain due to medical bills. Healthcare access outcomes included whether a patient had (1) a recent physician visit; (2) delayed medical care due to costs; and (3) needed medical care but did not get due to costs. Prescription medication outcomes (among those on medications) included (1) skipping prescription medication doses to save money; (2) taking less medication to save money; and (3) delaying filling medication prescription to save money, as well as (4) needing prescription medication but not getting due to cost. Financial strain outcomes included (1) problems paying medical for bills; (2) inability to pay for medical bills (among those with problems paying bills); and (3) worry about paying medical for bills if person were to get sick or have an accident. All outcomes were self-reported and adults were asked regarding their experiences within the last 1 year.

### Statistical analyses

We compared the baseline characteristics of adults age 64 years and 66 years using absolute standardized mean differences (aSMDs) or, for discrete variables with multiple categories, Mahalanobis distances [21]. We first examined the association between Medicare eligibility (age 64 versus 66 years) and outcomes using a linear probability model estimated by ordinary least squares regression with robust standard errors [22]. This analysis estimates the effect of Medicare eligibility on the outcome under the first component of the local randomization assumption described above.

To examine the effects of Medicare enrollment, we exploited the discontinuity in the probability of Medicare coverage around the eligibility cutoff (between individuals age 64 versus 66 years), in a local randomization-based regression discontinuity approach [17,23,24]. This approach is essentially an instrumental variable analysis that “rescales” the association of Medicare eligibility with each outcome by dividing it by the change in Medicare enrollment (between individuals age 64 versus 66 years). Specifically, we used 2-stage least squares regression [25] with Medicare eligibility as the instrument and Medicare enrollment as the “treatment” [26,27]. This analysis attempts to estimate the effect of Medicare enrollment on outcomes among individuals 64 or 66 years of age who are “compliers” to Medicare eligibility, that is, individuals who would enroll were they eligible for Medicare, and who would not enroll were they ineligible (this effect is sometimes referred to as a local average treatment effect or the effect among “compliers”). Interpreting this analysis as estimating the average treatment effect on compliers (around the cutoff) requires 3 assumptions, in addition to both components of the local randomization assumption: (1) that Medicare eligibility is associated with Medicare enrollment, which is verifiable from the data; (2) that the effect of Medicare eligibility on outcomes occurs exclusively through its effect on Medicare enrollment, an exclusion restriction assumption; and (3) that there are no individuals who would enroll were they ineligible and who would not enroll were they eligible for Medicare (a “no defiers” assumption) [26]. We chose to work with the local randomization-based regression discontinuity approach to study Medicare enrollment because it naturally extends our approach for studying the effect of Medicare eligibility, can account for the complex survey design of the NHIS, and can support statistical inference when age (the “running” variable in our analyses) only takes a small number of discrete values and when values close to the cutoff are not observed (in fact, our data has a “gap” at age 65 years because of discreteness in the reporting of age in NHIS) [28,29]. Continuity-based approaches for regression discontinuity methods may not perform well when there are no observations around the cutoff and cannot easily accommodate the complex survey design of the NHIS [28,29].

For all analyses of Medicare eligibility and enrollment, estimates reflect absolute percentage point differences in outcomes among adults age 64 versus 66 years. We performed these analyses separately for the low-income (≤300% FPL) and higher-income (>300% FPL) groups, and for both groups combined. Because there is a small change in the FPL at age 65 years (in 2017, $12,752 for an adult <65 years versus $11,756 for an adult 65 years or older) [30], we also evaluated outcomes just above and below the eligibility cutoff among adults with low versus higher levels of educational attainment (less than a Bachelor’s degree versus Bachelor’s degree or higher, respectively), because education is strongly associated with income and would not be expected to materially change from 64 to 66 years [31]. Nationally representative estimates and standard errors were obtained accounting for the NHIS survey design.

### Additional analyses

We performed several additional analyses to examine the stability of our main results. First, we repeated the main analyses after adjusting for self-reported clinical comorbidities (hypertension, hyperlipidemia, diabetes, myocardial infarction, stroke, chronic obstructive pulmonary disease, cancer). Second, we adjusted for employment status. Third, we adjusted for clinical comorbidities, employment status, and other baseline characteristics (e.g., education, region). Fourth, we used a more stringent definition to identify low-income adults—a household income of ≤200% FPL. Fifth, we widened the age groups around the Medicare eligibility threshold, to compare adults ages 63 to 64 years and adults ages 66 to 67 years. Finally, we also performed analyses using a continuity-based regression discontinuity approach to estimate the effect of Medicare eligibility at the cutoff of 65 years [32,33].

We excluded respondents with missing information on age, Medicare enrollment, and income (<0.5% of the total sample). In addition, individuals with missing responses were excluded in the analysis of each respective outcome (<1%). In the adjusted analyses, we also restricted to individuals with complete data on all the covariates included in each regression. The data underlying the results presented in the study are publicly available from the National Center for Health Statistics (https://www.cdc.gov/nchs/nhis/2019nhis.htm). Analyses were conducted with R version 4.0.3 and 4.1 and Stata version 16. Scripts to reproduce all analyses are available upon request to the Smith Center for Outcomes Research. We followed the RECORD reporting guidelines. This study did not have a prespecified protocol, and all additional analyses should be considered post hoc. NHIS has been determined to not require institutional review board approval by the National Bureau of Economics Research institutional review board.

## Results

### Baseline characteristics

From an unweighted sample of 31,997 individuals, baseline demographics and clinical characteristics of low-income adults ages 64 years (*n* = 1,660,118), compared to those age 66 years (*n* = 1,488,875), are shown in **Table 1**. The characteristics of these 2 populations were approximately balanced. In addition, baseline characteristics of higher-income adults age 64 years (*n* = 2,110,995) and 66 years (*n* = 2,167,676) were also largely similar.

**Table 1:** Baseline characteristics of the US population ages 64 and 66 years by income level^1^.

	Low-Income^2^			Higher-Income^3^			Overall		
	Age 64 years	Age 66 years	aSMD	Age 64 years	Age 66 years	aSMD	Age 64 years	Age 66 years	aSMD
**Weighted Population Size** ^**4**^	1,660,188	1,488,875		2,110,995	2,167,676		3,771,183	3,656,550	
**Insurance**			1.573			2.499			1.981
Medicare	27.6	87.8		8.0	85.9		16.6	86.7	
Other Public^5^	17.0	0		0.8	0		8.0	0	
Private or Other	38.9	9.6		85.4	13.4		64.9	11.9	
Uninsured	16.5	2.5		5.8	0.7		10.5	1.4	
**Female (%)**	59.2	59.7	0.009	50	49.4	0.012	54.1	53.6	0.010
**Race (%)**			0.22			0.1			0.142
White	59.1	64.9		82.9	83.5		72.4	76.0	
Black	17.0	13.2		5.3	5.7		10.4	8.8	
Hispanic	15.6	16.7		6.4	5.3		10.5	9.9	
Asian	4.7	4.3		3.4	4.3		4.0	4.3	
Other	3.5	0.9		2.0	1.1		2.7	1.0	
**Married**	48.0	46.2	0.037	78.4	76.4	0.048	65.1	64.2	0.019
**Education (Bachelor’s Degree or Higher)**	10.8	12.5	0.052	41.0	41.6	0.013	27.8	29.8	0.044
**Employment**			0.353			0.418			0.381
Not employed or retired	32.7	24.7		8.8	3.9		19.3	12.3	
Employed	32.2	23.1		60.5	46.3		48.1	36.9	
Retired	35.1	52.3		30.7	49.8		32.6	50.8	
**Self-Reported Comorbidities**									
Hypertension	67.5	60.3	0.151	47.7	44.4	0.066	56.4	50.9	0.111
Hyperlipidemia	46.8	46	0.016	39.9	39.5	0.008	42.9	42.2	0.016
Diabetes	28.2	24.6	0.082	12.2	11.6	0.019	19.2	16.9	0.061
Myocardial Infarction	9.5	7.3	0.078	3.8	2.0	0.107	6.3	4.2	0.096
Stroke	8.9	6.7	0.081	3.1	1.9	0.078	5.7	3.9	0.085
Chronic Obstructive Pulmonary Disease	15.7	8.2	0.234	5.6	3.8	0.083	10.0	5.6	0.166
Cancer	14.4	14.6	0.007	21.2	17.5	0.094	18.2	16.3	0.049
**US Region** ^**6**^			0.127			0.055			0.068
Midwest	22.6	19.7		20.4	21.2		21.4	20.6	
Northeast	15.5	12.5		21.3	19.3		18.7	16.5	
South	40.2	43.3		34.9	36.4		37.2	39.2	
West	21.7	24.5		23.4	23.1		22.6	23.7	

aSMD, absolute standardized mean difference; FPL, federal poverty level; NHIS, National Health Interview Survey.

We compared the distribution of the covariates between the 2 populations using aSMDs or, for discrete variables with multiple categories, Mahalanobis distances.

^1^Total unweighted sample size = 1,162. Adults missing information on age, Medicare enrollment, and income were excluded (<0.5%); information is presented for observations with available data for each covariate.

^2^Low-income was defined as family income ≤300% of the FPL.

^3^Higher-income was defined as family income >300% FPL.

^4^National estimates based on survey weights for the 2019 NHIS.

^5^For adults age 66 years, individuals who were dually enrolled in Medicare and Medicaid are included in the “Medicare” category rather than the “other public” category, consistent with the approach used by NHIS.

^6^US Census Bureau regions.

### Medicare enrollment

**Fig 1:**
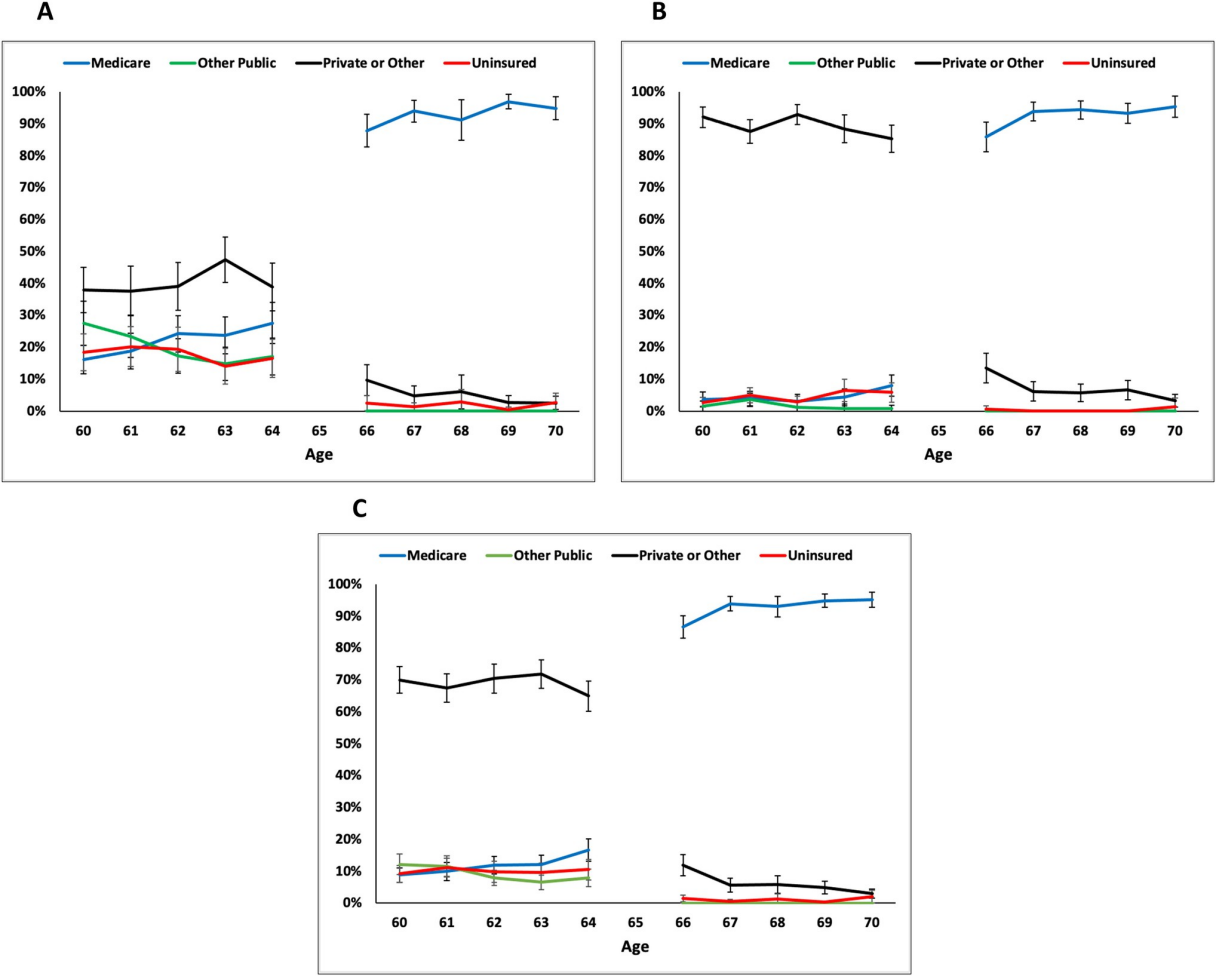
Percentage of US adults with health insurance coverage by income-level, age 60 to 70 years. The percentage of US adults covered by each type of health insurance. A large change in the percentage of US adults insured by Medicare is observed from age 64 to 66 years in the low-income (Panel A), higher-income (Panel B), and overall (Panel C) populations. (A) Low-income adults. (B) Higher-income adults. (C) Overall population.

There was a substantial difference in Medicare enrollment between low-income adults ages 64 and 66 years (27.6% versus 87.8%, *p* < 0.001) **(Table 1 and Fig 1A)**, and similar patterns were observed among higher-income adults (8.0% versus 85.9%, *p* < 0.001) **(Fig 1B)**. Overall trends in Medicare enrollment irrespective of income are displayed **(Fig 1C)**.

### Healthcare access by income

**Fig 2:**
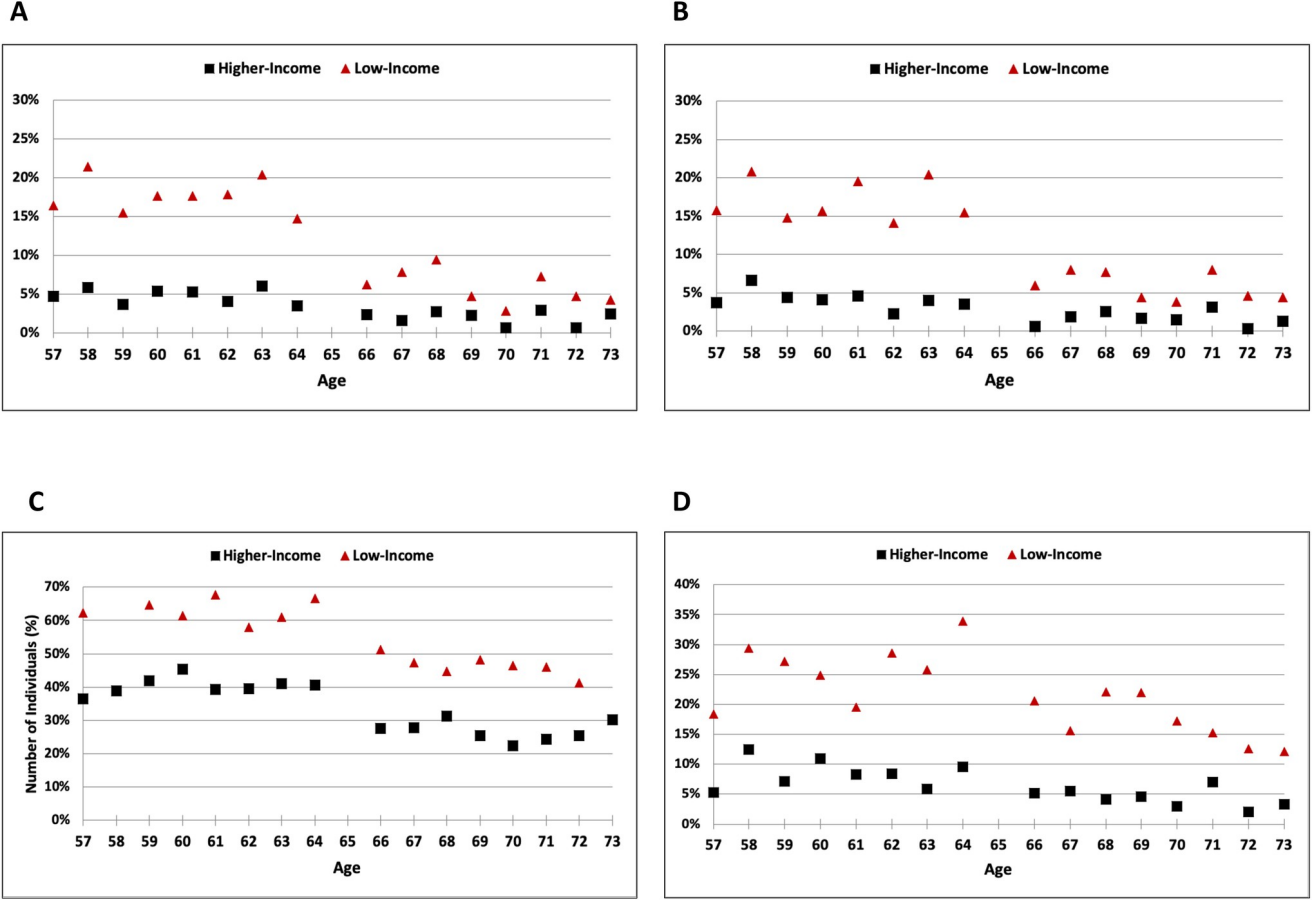
Measures of access and affordability by income level, age 57 to 73 years. The percentage of US adults who reported (A) delaying medical care due to cost, (B) not seeking medical care due to cost, (C) worrying about medical bills, and (D) experiencing problems paying medical bills. Remaining outcomes are shown in Figs A-F in **S1 Text**. (A) Delayed medical care due to cost. (B) Did not seek medical care due to cost. (C) Get sick or have accident, worry about paying medical bills. (D) Problems paying medical bills.

In the low-income population, Medicare eligibility was associated with a decrease in the percentage of adults who delayed medical care due to costs (14.7% [age 64 years] versus 6.2% [66 years]; −8.5% [95% CI −14.7%, −2.4%], *P* = 0.007) and did not seek medical care due to costs (15.5% versus 5.9%; −9.6% [−15.9%, −3.2%], *P* = 0.003) **(Fig 2A and 2B)**. There was a small decrease in the percentage with recent physician visits, although this association was not statistically significant (93.6% versus 89.4%: −4.2% [−10.1%, 1.7%], *P* = 0.16) **(Table 2 and Fig A in S1 Text)**. Among higher-income adults, Medicare eligibility was also associated with a significant decrease in not seeking medical care due to costs (3.5% versus 0.6%; −2.9% [−5.3%, −0.5%], *P* = 0.02), while delays in medical care due to costs (3.5% versus 2.4%; −1.1% [−4.0%, 1.7%], *P* = 0.44) and recent physician visits (91.7% versus 93.8%; 2.1% [−2.7%, 6.9%], *P* = 0.78) did not show a significant association.

**Table 2:** Association of Medicare eligibility and enrollment with healthcare access by income level^1^.

		Population Size^2^	Adults Age 64 years	Adults Age 66 years	Effect of Medicare Eligibility^3^	*p*-Value	Effect of Medicare Enrollment^3^	*p*-Value
**Recent Physician Visit**	**Low-Income**	3,141,300	93.6%	89.4%	−4.2% (−10.1%, 1.7%)	0.16	−7.0% (−16.8%, 2.8%)	0.16
	**Higher- Income**	4,274,102	91.7%	93.8%	2.1% (−2.7%, 6.9%)	0.39	2.7% (−3.4%, 8.8%)	0.38
	**Overall**	7,415,402	92.5%	92.0%	−0.5% (−4.2%, 3.2%)	0.78	−0.8% (−6.0%, 4.5%)	0.78
**Delayed Medical Care Due to Cost**	**Low-Income**	3,139,409	14.7%	6.2%	−8.5% (−14.7%, −2.4%)	0.007	−14.2% (−24.7%, −3.7%)	0.008
	**Higher-Income**	4,276,153	3.5%	2.4%	−1.1% (−4.0%, 1.7%)	0.44	−1.4% (−5.1%, 2.2%)	0.44
	**Overall**	7,415,561	8.4%	3.9%	−4.5% (−7.7%, −1.3%)	0.005	−6.4% (−11.0%, −1.8%)	0.006
**Did Not Seek Medical Care Due to Cost**	**Low-Income**	3,139,409	15.5%	5.9%	−9.6% (−15.9%, −3.2%)	0.003	−15.9% (−26.9%, −4.9%)	0.005
	**Higher-Income**	4,276,153	3.5%	0.6%	−2.9% (−5.3%, −0.5%)	0.02	−3.7% (−6.8%, −0.6%)	0.02
	**Overall**	7,415,561	8.8%	2.8%	−6.0% (−9.1%, −2.9%)	<0.001	−8.6% (−13.0%, −4.1%)	<0.001

^1^Adults with a missing response were excluded from the analysis of the respective outcome (<1% across all outcomes).

^2^National estimates based on survey weights for the 2019 National Health Interview Survey.

^3^Percentage point change in outcomes.

Medicare enrollment was associated with a decrease in the percentage of low-income adults who delayed medical care (−14.2%; [95% CI: −24.7, −3.7], *P* = 0.008) or did not seek medical care (−15.9%; [−26.9, −4.9], *P* = 0.005) due to costs **(Table 2)**. Among higher-income adults, Medicare enrollment was also associated with a decrease in not seeking medical care due to costs (−3.7%; [−6.8, −0.6], *P* = 0.02) and was not strongly associated with delays in medical care (−1.4%; [−5.1, 2.2], *P* = 0.44).

### Prescription medications by income

Medicare eligibility was not significantly associated with a change in the percentage of low-income adults who delayed filling a prescription to save money (12.1% versus 8.2%; −3.9% [95% CI −11.2%, 3.3%], *P* = 0.29), did not obtain a needed prescription due to cost (14.9% versus 9.9%; −5.0% [−12.3%, 2.3%], *P* = 0.18), skipped medication to save money (9.7% versus 9.1%; −0.6% [−8.0%, 6.8%], *P* = 0.87), or took less medication to save money (13.9% versus 9.1%; −4.8% [−12.8%, 3.2%], *P* = 0.24) **(Table 3)**. These patterns were similar in the higher-income group.

**Table 3:** Association of Medicare eligibility and enrollment with prescription medications by income level^1^.

		Population Size^2^	Adults Age 64 years	Adults Age 66 years	Effect of Medicare Eligibility^4^	*p*-Value	Effect of Medicare Enrollment^4^	*p*-Value
**Delayed Filling Prescription to Save Money** ^**3**^	**Low-Income**	2,601,998	12.1%	8.2%	−3.9% (−11.2%, 3.3%)	0.29	−6.8% (−19.3%, 5.7%)	0.29
	**Higher-Income**	3,708,473	5.3%	3.5%	−1.8% (−5.9%, 2.3%)	0.39	−2.3% (−7.6%, 2.9%)	0.39
	**Overall**	6,310,471	8.2%	5.3%	−2.8% (−6.6%, 0.9%)	0.14	−4.1% (−9.5%, 1.3%)	0.14
**Needed Prescription Medication but Did Not Get It Due to Cost**	**Low-Income**	3,135,558	14.9%	9.9%	−5.0% (−12.3%, 2.3%)	0.18	−8.3% (−20.6%, 4.0%)	0.18
	**Higher-Income**	4,276,153	4.3%	3.9%	−0.4% (−3.8%, 3.0%)	0.82	−0.5% (−4.9%, 3.9%)	0.82
	**Overall**	7,411,711	9.0%	6.3%	−2.6% (−6.4%, 1.2%)	0.18	−3.7% (−9.2%, 1.7%)	0.18
**Skipped Medication Doses to Save Money** ^**3**^	**Low-Income**	2,601,998	9.7%	9.1%	−0.6% (−8.0%, 6.8%)	0.87	−1.1% (−13.8%, 11.6%)	0.87
	**Higher-Income**	3,708,473	5.0%	2.6%	−2.3% (−6.4%, 1.7%)	0.25	−3.0% (−8.2%, 2.2%)	0.25
	**Overall**	6,310,471	7.0%	5.2%	−1.8% (−5.6%, 2.0%)	0.36	−2.6% (−8.0%, 2.9%)	0.36
**Took Less Medication to Save Money** ^**3**^	**Low-Income**	2,601,998	13.9%	9.1%	−4.8% (−12.8%, 3.2%)	0.24	−8.3% (−22.2%, 5.6%)	0.24
	**Higher-Income**	3,708,473	2.9%	2.2%	−0.7% (−3.3%, 1.9%)	0.60	−0.9% (−4.3%, 2.5%)	0.60
	**Overall**	6,310,471	7.6%	5.0%	−2.6% (−6.4%, 1.2%)	0.17	−3.8% (−9.3%, 1.7%)	0.17

^1^Adults with a missing response were excluded from the analysis of the respective outcome (<1% across all outcomes).

^2^National estimates based on survey weights for the 2019 National Health Interview Survey.

^3^Among adults taking prescription medications.

^4^Percentage point change in outcomes.

Medicare enrollment was not significantly associated with a change in the percentage of low-income adults who delayed filling a prescription to save money (−6.8%; [95% CI: −19.3, 5.7], *P* = 0.29), did not obtain a necessary prescription due to cost (−8.3%; [95% CI: −20.6, 4.0], *P* = 0.18), skipped medication (−1.1%; [95% CI: −13.8, 11.6], *P* = 0.87), or took less medication (−8.3%; [95% CI: −22.2, 5.6], *P* = 0.24) to save money **(Table 3)**. These patterns were similar in the higher-income group.

### Financial strain by income

For measures of financial strain, Medicare eligibility in the low-income population was associated with large decreases in the percentage of adults who worried about paying medical bills (66.5% versus 51.1%; −15.4% [95% CI −25.4%, −5.4%], *P* = 0.003), had problems paying medical bills (33.9% versus 20.6%; −13.3% [−23.0%, −3.6%], *P* = 0.007), or were unable to pay for medical bills (77.3% versus 53.4%; −23.9% [−44.7%, −3.0%], *P* = 0.03), **(Table 4 and Fig 2)**. In the higher-income group, eligibility was also associated more modest decreases in the percentage who were worried about paying medical bills (40.5% versus 27.5%; −13.0% [−21.4%, −4.5%], *P* = 0.003) and had problems paying medical bills (9.5% versus 5.2%; −4.3% [−8.8%, 0.1%], *P* = 0.05), but was not significantly associated with the percentage who were unable to pay for medical bills (38.9% versus 43.1%; 4.2% [−30.0%, 38.4%], *P* = 0.81).

**Table 4:** Association of Medicare eligibility and enrollment with financial strain due to medical bills by income level^1^.

		Population Size^2^	Adults Age 64 years	Adults Age 66 years	Effect of Medicare Eligibility^3^	*p*-Value	Effect of Medicare Enrollment^3^	*p*-Value
**Get Sick or Have Accident, Worry about Paying Medical Bills**	**Low-Income**	3,138,787	66.5%	51.1%	−15.4% (−25.4%, −5.4%)	0.003	−25.6% (−42.5%, −8.7%)	0.003
	**Higher-Income**	4,273,658	40.5%	27.5%	−13.0% (−21.4%, −4.5%)	0.003	−16.7% (−27.7%, −5.6%)	0.003
	**Overall**	7,412,444	51.9%	37.2%	−14.8% (−21.8%, −7.8%)	<0.001	−21.1% (−31.4%, −10.9%)	<0.001
**Problems Paying Medical Bills**	**Low-Income**	3,141,300	33.9%	20.6%	−13.3% (−23.0%, −3.6%)	0.007	−22.1% (−38.5%, −5.7%)	0.008
	**Higher-Income**	4,260,352	9.5%	5.2%	−4.3% (−8.8%, 0.1%)	0.05	−5.5% (−11.2%, 0.1%)	0.06
	**Overall**	7,401,652	20.3%	11.4%	−8.8% (−14.1%, −3.5%)	0.001	−12.6% (−20.2%, −4.9%)	0.001
**Unable, Pay Medical Bills** ^**4**^	**Low-Income**	866,176	77.3%	53.4%	−23.9% (−44.7%, −3.0%)	0.03	−44.5% (−85.1%, −3.9%)	0.03
	**Higher-Income**	310,837	38.9%	43.1%	4.2% (−30.0%, 38.4%)	0.81	5.3% (−37.8%, 48.3%)	0.81
	**Overall**	1,177,013	67.2%	50.7%	−16.6% (−35.1%, 2.0%)	0.08	−27.4% (−58.1%, 3.3%)	0.08

^1^Adults with a missing response were excluded from the analysis of the respective outcome (<1% across all outcomes).

^2^National estimates based on survey weights for the 2019 National Health Interview Survey.

^3^Percentage point change in outcomes.

^4^Among adults reporting problems paying medical bills.

Medicare enrollment was associated with a large decrease in the percentage of low-income adults who were likely to worry about paying medical bills (−25.6%; [−42.5%, −8.7%], *P* = 0.003), had problems paying medical bills (−22.1% [95% CI: −38.5%, −5.7%], *P* = 0.008), and were unable to pay for medical bills (−44.5%; [−85.1%, −3.9%], *P* = 0.03) **(Table 4)**. Among higher-income adults, enrollment was also associated with more modest decreases in the percentage of adults who worried about paying medical bills (−16.7%; [−27.7%, −5.6%], *P* = 0.003), experienced problems paying bills (−5.5%; [−11.2%, 0.1%], *P* = 0.06), and was not significantly associated with being unable to pay for medical bills (5.3%; [−37.8%, 48.3%], *P* = 0.81).

### Access, prescription medications, and financial strain by educational attainment

Baseline characteristics of adults with low and higher educational attainment are shown in **Table A in S1 Text**. Among adults with low educational attainment, Medicare eligibility was associated with a decrease in delaying medical care due to cost (−4.7%; [−8.9%, −0.4%], *P* = 0.03) and not seeking medical care due to cost (−7.0%; [−11.2%, −2.8%], *P* = 0.02), while more modest associations were observed among adults with higher education **(Table 5).** There were no significant associations between eligibility and measures of prescription drug affordability among adults with low or higher education. For measures of financial strain, there were large decreases in worrying about paying for medical bills (−16.1%; [−24.4%, −7.9%], *P* < 0.001), problems paying medical bills (−9.4%; [−16.3%, −2.6%], *P* = 0.007), and inability to pay medical bills (−25.9%; [−45.9%, −6.0%], *P* = 0.01), although these associations were more modest and less consistent among adults with higher levels of education. The association between Medicare enrollment and all outcomes are shown in **Table 5**.

**Table 5:** Association of Medicare eligibility and enrollment at age 65 years on healthcare access, prescription medications, and financial strain by education level^1^.

		Population Size^2^	Adults Age 64 years	Adults Age 66 years	Effect of Medicare Eligibility^3^	*P*-Value	Effect of Medicare Enrollment^3^	*P*-Value
**Healthcare Access**								
**Recent Physician Visit**	**Low Education**	5,234,801	92.9%	91.8%	−1.1% (−5.8% to 3.6%)	0.64	−1.7% (−8.8% to 5.4%)	0.64
	**Higher Education**	2,117,250	91.5%	93.1%	1.6% (−4.4% to 7.5%)	0.60	2.0% (−5.4% to 9.4%)	0.60
	**Overall**	7,352,051	92.6%	92.2%	−0.3% (−4.0% to 3.3%)	0.86	−0.5% (−5.8% to 4.8%)	0.86
**Delayed Medical Care Due to Cost**	**Low Education**	5,232,909	9.9%	5.2%	−4.7% (−8.9% to −0.4%)	0.03	−7.1% (−13.7% to −0.6%)	0.03
	**Higher Education**	2,119,301	4.5%	0.9%	−3.6% (−6.6% to −0.6%)	0.02	−4.5% (−8.3% to −0.7%)	0.02
	**Overall**	7,352,210	8.4%	3.9%	−4.5% (−7.7% to −1.3%)	0.006	−6.4% (−11.0% to −1.8%)	0.007
**Did Not Seek Medical Care Due to Cost**	**Low Education**	5,232,909	10.9%	3.9%	−7.0% (−11.2% to −2.8%)	0.001	−10.7% (−17.3% to −4.1%)	0.002
	**Higher Education**	2,119,301	3.3%	0.3%	−3.0% (−5.6% to −0.5%)	0.02	−3.7% (−6.9% to −0.6%)	0.02
	**Overall**	7,352,210	8.7%	2.8%	−6.0% (−9.0% to −2.9%)	<0.001	−8.5% (−13.1% to −4.0%)	<0.001
**Prescription Medications**								
**Delayed Filling Prescription to Save Money** ^**3**^	**Low Education**	4,484,967	7.4%	5.6%	−1.8% (−5.8% to 2.2%)	0.37	−2.8% (−9.0% to 3.4%)	0.37
	**Higher Education**	1,777,230	7.1%	4.7%	−2.4% (−9.0% to 4.2%)	0.47	−3.0% (−11.1% to 5.2%)	0.47
	**Overall**	6,262,196	7.3%	5.3%	−2.0% (−5.4% to 1.4%)	0.25	−2.9% (−7.8% to 2.0%)	0.25
**Needed Prescription Medication but Did Not Get It Due to Cost**	**Low Education**	5,229,059	9.0%	6.9%	−2.1% (−6.4% to 2.3%)	0.35	−3.2% (−9.9% to 3.5%)	0.35
	**Higher Education**	2,119,301	5.9%	5.1%	−0.8% (−6.1% to 4.6%)	0.78	−1.0% (−7.6% to 5.7%)	0.78
	**Overall**	7,348,359	8.1%	6.4%	−1.7% (−5.3% to 1.8%)	0.33	−2.5% (−7.6% to 2.6%)	0.33
**Skipped Medication Doses to Save Money** ^**3**^	**Low Education**	4,484,967	5.7%	5.2%	−0.5% (−4.4% to 3.4%)	0.81	−0.8% (−6.8% to 5.3%)	0.81
	**Higher Education**	1,777,230	7.3%	5.3%	−2.0% (−8.6% to 4.5%)	0.54	−2.5% (−10.6% to 5.5%)	0.54
	**Overall**	6,262,196	6.1%	5.2%	−0.9% (−4.3% to 2.5%)	0.59	−1.3% (−6.2% to 3.5%)	0.59
**Took Less Medication to Save Money** ^**4**^	**Low Education**	4,484,967	7.8%	5.5%	−2.3% (−6.6% to 2.0%)	0.30	−3.6% (−10.3% to 3.2%)	0.30
	**Higher Education**	1,777,230	4.1%	3.7%	−0.3% (−5.2% to 4.5%)	0.89	−0.4% (−6.4% to 5.6%)	0.89
	**Overall**	6,262,196	6.7%	5.0%	−1.8% (−5.2% to 1.6%)	0.30	−2.6% (−7.5% to 2.3%)	0.30
**Financial Strain Due to Medical Bills**								
**Get Sick or Have Accident, Worry about Paying Medical Bills**	**Low Education**	5,232,287	57.1%	40.9%	−16.1% (−24.4% to −7.9%)	<0.001	−24.6% (−37.7% to −11.6%)	<0.001
	**Higher Education**	2,116,806	37.8%	27.7%	−10.2% (−21.1% to 0.8%)	0.07	−12.6% (−26.4% to 1.2%)	0.07
	**Overall**	7,349,093	51.7%	37.0%	−14.7% (−21.7% to −7.8%)	<0.001	−21.1% (−31.3% to −10.9%)	<0.001
**Problems Paying Medical Bills**	**Low Education**	5,234,801	23.5%	14.0%	−9.4% (−16.3% to −2.6%)	0.007	−14.4% (−25.0% to −3.8%)	0.008
	**Higher Education**	2,103,500	10.0%	5.4%	−4.5% (−10.9% to 1.9%)	0.16	−5.5% (−13.4% to 2.4%)	0.17
	**Overall**	7,338,300	19.7%	11.5%	−8.3% (−13.5% to −3.1%)	0.002	−11.8% (−19.3% to −4.3%)	0.002
**Unable to Pay Medical Bills** ^**5**^	**Low Education**	986,345	73.4%	47.5%	−25.9% (−45.9% to −6.0%)	0.01	−47.6% (−85.1% to −10.1%)	0.01
	**Higher Education**	160,324	18.9%	70.0%	51.1% (13.0% to 89.2%)	0.009	57.9% (12.0% to 103.8%)	0.01
	**Overall**	1,146,669	65.9%	50.7%	−15.2% (−33.8% to 3.5%)	0.11	−25.7% (−57.2% to 5.9%)	0.11

^1^Adults with a missing response were excluded from the analysis of the respective outcome (<1% across all outcomes).

^2^National estimates based on survey weights for the 2019 National Health Interview Survey.

^3^Percentage point change in outcomes.

^4^Among adults taking prescription medications.

^5^Among adults reporting problems paying medical bills.

### Additional analyses

Overall, our sensitivity analyses were consistent with the main income-based analyses reported above. First, we repeated our main analysis after adjusting for clinical comorbidities and found associations of similar direction as our main results, although estimates were somewhat attenuated (**Table B in S1 Text**). Second, we repeated our analysis after controlling for employment status, which resulted in findings that were also similar to our main analysis (**Table C in S1 Text**). Third, after we adjusted for clinical comorbidities, employment, and other baseline characteristics, there were statistically significant decreases in the percentage of low-income adults who worried about paying medical bills or did not seek medical care due to costs **(Table D in S1 Text)**. Fourth, we used a different definition to identify low-income adults in the US (family income ≤200% FPL), and our findings were consistent with our main analysis **(Table E in S1 Text)**. Fifth, we expanded the age groups to age 63 to 64 years versus 66 to 67 years **(Table F in S1 Text)**, which also resulted in consistent findings **(Table G in S1 Text)**. We also repeated these additional analyses by educational attainment **(Tables H-J in S1 Text).** Finally, regression discontinuity analyses (by income status) are presented in **Tables K and L in S1 Text.**

## Discussion

We found that Medicare eligibility and enrollment at age 65 years were associated with increases in measures of healthcare access, reductions in financial strain due to medical bills, and no change in cost-related barriers to prescription medication use among low-income adults in the US. These associations were more modest and less consistent among higher-income adults when compared with the low-income population. The overall patterns we observed in the income-based analyses were similar when evaluating the association of Medicare with outcomes by levels of educational attainment. Overall, our findings provide contemporary insights on the impact of Medicare by socioeconomic status in the ACA era and have important implications for the ongoing policy discussions about expansion of the Medicare program.

Over the 2 years, the debate on whether to expand Medicare has intensified among policymakers, including recent proposals to lower the Medicare eligibility age from 65 to 60 years [3,4]. Our findings provide insights on how this shift in policy might influence the affordability of healthcare for low-income Americans. For example, we found that approximately 1 in 6 low-income adults (age 64 years) were unable to seek healthcare due to cost and that a similar share delayed medical care due to cost. These financial barriers in healthcare access were mitigated by Medicare eligibility and enrollment. In addition, nearly 70% of low-income adults worried about paying their medical bills, and Medicare enrollment was associated with a marked reduction in the percentage of adults who experienced this type of financial strain. Notably, we did not observe changes in cost-related barriers to prescription medication use associated with Medicare eligibility or enrollment, which could potentially reflect the financial burden of out-of-pocket costs for medications among Medicare beneficiaries [14].

Our findings have implications for policymakers weighing strategies to improve health equity in the US [7,34–36]. Low-income adults have a higher burden of chronic disease and worse health outcomes than their higher-income counterparts [5], in part because they disproportionately face financial barriers in healthcare access and struggle to afford prescription drugs [37]. Moreover, uninsured rates have increased for low-income adults over the last few years, and even those with coverage are increasingly experiencing “underinsurance” [9,38], leaving them susceptible to catastrophic expenditures and negative wealth shocks that may be detrimental to health [39]. Our findings suggest that reducing the eligibility age of Medicare may help improve equity, by increasing healthcare access and reducing financial strain for this vulnerable subset of the population.

We also found that higher-income adults were less likely to face challenges paying for healthcare services than their low-income counterparts. However, this group still experienced increases in some measures of healthcare access as well as a reduction in worrying about paying medical bills, even though the vast majority were privately insured prior to enrollment. Over the last decade, average premiums for employer-sponsored private insurance plans have increased [40], and the average deductible for these plans has more than doubled [38]. As a result, working-age American face rising out-of-pocket costs. Our finding that Medicare coverage is associated with reductions in worrying about paying for medical bills—even among high-income adults—is important, given escalating concerns about a “crisis in affordability” for private insurance coverage in the US [9]. As policymakers consider expansion of the Medicare program, understanding how changes to the program may differentially impact low- and higher-income adults will be necessary to improve health equity.

Our analysis extends upon prior work that has examined the implications of expanding Medicare by focusing on measures of access, affordability, and financial strain in a contemporary population of low-income adults. Using data from 1999 to 2003, one prior study found that the onset of Medicare eligibility led to increases in health insurance coverage and utilization, and a subsequent analysis found that Medicare was associated with improved mortality in patients hospitalized for acute conditions [11,41]. Another analysis, in 2004, reported that universal healthcare at age 65 years was associated with reductions in disparities for lower-income adults [42]. More recent work has shown that Medicare eligibility improves access and affordability [12], reduces medical debt [43], decreases out-of-pocket spending [44], reduces catastrophic expenditures for patients hospitalized with surgical conditions [45], and also reduces racial disparities in insurance coverage, access to care, and self-reported health [46]. Our work extends upon this evidence by examining the effects of Medicare eligibility specifically by income status (low- versus higher-income), while also estimating the effects of Medicare enrollment, in a contemporary, post-ACA population of US adults. In addition, we focus on a broad and comprehensive set of measures of healthcare access, financially strain (e.g., worry about paying medical bills, problems paying medical bills), and cost-related barriers to medication use (e.g., delayed or skipped medication to safe money), which reflect patients’ perspectives and experience, and have largely not been examined by income status in prior work. Given increasing income-based inequities in health in the US, and rapid changes in the healthcare landscape over the past decade (e.g., passing of the ACA, expansion of the Medicaid program, rising “underinsurance”), our study provides novel and timely insights on how Medicare eligibility and enrollment impact a contemporary population of low-income adults. As policymakers’ debate whether to reduce the age of eligibility of Medicare, our findings suggest that doing so may lead to reductions in income-based inequities in healthcare in the US [34].

This study has several limitations. First, an assumption of our analysis is that adults age 64 and 66 years are similar except for Medicare eligibility and its effects. We found that most baseline characteristics were similar between these age groups. Nonetheless, our findings generally remained consistent after performing several stability analyses, including repeating the main analysis after adjustment for comorbidities, employment status, and other characteristics (e.g., education), using a more stringent definition of low-income (≤200% FPL), and expanding age windows around the Medicare eligibility threshold. Second, we observed similar patterns when evaluating outcomes among adults with low versus higher levels of educational attainment, which does not drastically change around age 65 years and is strongly associated with income and socioeconomic status. Third, our results can be interpreted as comparing Medicare enrollment versus a distribution of alternative insurance options (as observed in our data) and our estimates may not apply to a population with a different distribution of non-Medicare coverage options. The extent to which the effect we estimate is determined by different types of insurance transitions at age 65 years is an important area for further research. Fourth, the observational nature of our analyses makes any causal interpretation of our results, especially those for Medicare enrollment, dependent on untestable assumptions. Our empirical findings, however, document large differences in some outcomes between 2 nationally representative groups of individuals with similar—albeit not identical—age, just above and below the Medicare eligibility age cutoff. These findings suggest that changes in outcomes may be driven—at least in part—by the shift in the distribution of insurance coverage and, in particular, the large increase in Medicare coverage that occurs around the Medicare eligibility cutoff. More definitively ruling out alternative explanations would require additional, ideally experimental, research. Fifth, because NHIS does not reliably capture information about dual enrollment in Medicare and Medicaid, we were unable to account for dual enrollment in our analysis [47].

## Conclusions

Medicare eligibility and enrollment at age 65 years was associated with improvements in some measures of healthcare access and the financial strain, and no change in cost-related barriers to prescription medication use, among low-income adults in the US. Higher-income adults also experienced some improvements, although associations were less strong. As the debate on whether to expand Medicare continues, policymakers should consider the potential implications of doing so on healthcare access and affordability for low-income adults and, more broadly, health equity in the US.

## Supporting information

S1 RECORD Checklist Record checklist. (DOCX)

S1 Text Supporting tables and figures. **Table A.** Baseline characteristics of US populations age 64 and 66 years by educational attainment. **Table B.** Association of Medicare eligibility and enrollment on healthcare access, prescription medications, and financial strain after adjusting for self-reported comorbidities. **Table C.** Association of Medicare eligibility and enrollment on healthcare access, prescription medications, and financial strain by income level after adjusting for employment. **Table D.** Association of Medicare eligibility and enrollment on healthcare access, prescription medications, and financial strain after adjusting for self-reported comorbidities, employment, other baseline characteristics. **Table E.** Association of Medicare eligibility and enrollment on healthcare access, prescription medications, and financial strain by income level using: alternative definition for low-income population (≤200% FPL). **Table F.** Baseline characteristics of the US population ages 63–64 and 66–67 years by income level. **Table G.** Association of Medicare eligibility and enrollment on healthcare access, prescription medications, and financial strain by income level using wider age ranges (63–64 years vs. 66–67 years). **Table H.** Association of Medicare eligibility and enrollment on healthcare access, prescription medications, and financial strain by education level after adjusting for employment. **Table I.** Association of Medicare eligibility and enrollment on healthcare access, prescription medications, and financial strain by education level after adjusting for self-reported clinical comorbidities. **Table J.** Association of Medicare eligibility and enrollment on healthcare access, prescription medications, and financial strain by education level after adjusting for self-reported comorbidities, employment, other baseline characteristics. **Table K.** Association of Medicare eligibility on healthcare access, prescription medications, and financial strain by income level—Continuity-based regression discontinuity analysis with 3-year bandwidth. **Table L.** Association of Medicare eligibility on healthcare access, prescription medications, and financial strain by income level—Continuity-based regression discontinuity analysis with 8-year bandwidth. **Fig A.** Measures of access and affordability by income level, recent physician visit, age 57 to 73 years. **Fig B.** Measures of access and affordability by income level, delayed filling prescription to save money, age 57 to 73 years. **Fig C.** Measures of access and affordability by income level, needed prescription medication but did not get it due to cost, age 57 to 73 years. **Fig D.** Measures of access and affordability by income level, skipped medication doses to save money, age 57 to 73 years. **Fig E.** Measures of access and affordability by income level, took less medication to save money, age 57 to 73 years. **Fig F.** Measures of access and affordability by income level, unable to pay medical bills, age 57 to 73 years.
(DOCX)

## Abbreviations

ACA: Affordable Care Act
aSMD: absolute standardized mean difference
FPL: federal poverty level
NHIS: National Health Interview Survey
