# Supplementary material for: Medicare eligibility and healthcare access, affordability, and financial strain for low- and higher-income adults in the United States: A regression discontinuity analysis
Source: PLoS Med. 2022 Oct 4;19(10):e1004083. doi: 10.1371/journal.pmed.1004083 (PMC9531792; doi:10.1371/journal.pmed.1004083)
Supplement: S1 Text — Table A. Baseline characteristics of US populations age 64 and 66 years by educational attainment. Table B. Association of Medicare eligibility and enrollment on healthcare access, prescription medications, and financial strain after adjusting for self-reported comorbidities. Table C. Association of Medicare eligibility and enrollment on healthcare access, prescription medications, and financial strain by income level after adjusting for employment. Table D. Association of Medicare eligibility and enrollment on healthcare access, prescription medications, and financial strain after adjusting for self-reported comorbidities, employment, other baseline characteristics. Table E. Association of Medicare eligibility and enrollment on healthcare access, prescription medications, and financial strain by income level using: alternative definition for low-income population (≤200% FPL). Table F. Baseline characteristics of the US population ages 63–64 and 66–67 years by income level. Table G. Association of Medicare eligibility and enrollment on healthcare access, prescription medications, and financial strain by income level using wider age ranges (63–64 years vs. 66–67 years). Table H. Association of Medicare eligibility and enrollment on healthcare access, prescription medications, and financial strain by education level after adjusting for employment. Table I. Association of Medicare eligibility and enrollment on healthcare access, prescription medications, and financial strain by education level after adjusting for self-reported clinical comorbidities. Table J. Association of Medicare eligibility and enrollment on healthcare access, prescription medications, and financial strain by education level after adjusting for self-reported comorbidities, employment, other baseline characteristics. Table K. Association of Medicare eligibility on healthcare access, prescription medications, and financial strain by income level—Continuity-based regression discontinuity analysis with [file pmed.1004083.s002.docx]

**S1 Text**

**Table A.** Baseline Characteristics of U.S. Population Age 64 and 66 Years by Educational Attainment

**Table B:** Association of Medicare Eligibility and Enrollment on Health Care Access, Prescription Medications, and Financial Strain After Adjusting for Self-Reported Comorbidities

**Table C:** Association of Medicare Eligibility and Enrollment on Health Care Access, Prescription Medications, and Financial Strain by Income Level after Adjusting for Employment

**Table D.** Association of Medicare Eligibility and Enrollment on Health Care Access, Prescription Medications, and Financial Strain After Adjusting for Self-Reported Comorbidities, Employment, other Baseline Characteristics

**Table E:** Association of Medicare Eligibility and Enrollment on Health Care Access, Prescription Medications, and Financial Strain by Income Level Using: Alternative Definition for Low-Income Population (≤200% FPL)

**Table F:** Baseline Characteristics of the U.S. Population Ages 63-64 and 66-67 Years by Income Level

**Table G:** Association of Medicare Eligibility and Enrollment on Health Care Access, Prescription Medications, and Financial Strain by Income Level Using Wider Age Ranges (63-64 years vs. 66-67 years)

**Table H.** Association of Medicare Eligibility and Enrollment on Health Care Access, Prescription Medications, and Financial Strain by Education Level After Adjusting for Employment

**Table I.** Association of Medicare Eligibility and Enrollment on Health Care Access, Prescription Medications, and Financial Strain by Education Level After Adjusting for Self-Reported Clinical Comorbidities

**Table J.** Association of Medicare Eligibility and Enrollment on Health Care Access, Prescription Medications, and Financial Strain by Education Level After Adjusting for Self-Reported Comorbidities, Employment, other Baseline Characteristics

**Table K.** Association of Medicare Eligibility on Health Care Access, Prescription Medications, and Financial Strain by Income Level – Continuity-Based Regression Discontinuity Analysis with 3-Year Bandwidth

**Table L.** Association of Medicare Eligibility on Health Care Access, Prescription Medications, and Financial Strain by Income Level – Continuity-Based Regression Discontinuity Analysis with 8-Year Bandwidth

**Fig A:** Measures of Access and Affordability by Income-Level, Recent Physician Visit, Age 57 to 73 Years

**Fig B:** Measures of Access and Affordability by Income-Level, Delayed Filling Prescription to Save Money, Age 57 to 73 Years

**Fig C:** Measures of Access and Affordability by Income-Level, Needed Prescription Medication but Did Not Get it Due to Cost, Age 57 to 73 Years

**Fig D:** Measures of Access and Affordability by Income-Level, Skipped Medication Doses to Save Money, Age 57 to 73 Years

**Fig E:** Measures of Access and Affordability by Income-Level, Took Less Medication to Save Money, Age 57 to 73 Years

**Fig F:** Measures of Access and Affordability by Income-Level, Unable to Pay Medical Bills, Age 57 to 73 Years

**Table A. Baseline Characteristics of U.S. Populations Age 64 and 66 Years by Educational Attainment**

|  | **Low Educational Attainment^1^** | | | **Higher Educational Attainment^2^** | | |
| --- | --- | --- | --- | --- | --- | --- |
|  | **Age 64 years** | **Age 66 years** | **aSMD** | **Age 64 years** | **Age 66 years** | **aSMD** |
| **Population Size^3^** | 2682750 | 2559813 |  | 1034379 | 1087439 |  |
| **Female (%)** | 54 | 55.8 | 0.038 | 57.1 | 48.7 | 0.168 |
| **Race (%)** |  |  | 0.181 |  |  | 0.188 |
| White | 70.5 | 73.4 |  | 79.6 | 82.6 |  |
| Black | 12.3 | 8.9 |  | 6.1 | 7.7 |  |
| Hispanic | 10.8 | 12.2 |  | 6.7 | 4.7 |  |
| Asian | 3.1 | 4 |  | 6.6 | 5 |  |
| Other | 3.4 | 1.4 |  | 0.9 | 0 |  |
| **Married** | 63 | 60.6 | 0.048 | 69.4 | 73.2 | 0.084 |
| **Employment** |  |  | 0.392 |  |  | 0.372 |
| Not employed, not retired | 22.6 | 15.2 |  | 11.8 | 4.5 |  |
| Employed | 44.6 | 33.1 |  | 54.3 | 46.3 |  |
| Retired | 32.8 | 51.7 |  | 33.9 | 49.2 |  |
| **Insurance** |  |  | 1.763 |  |  | 2.739 |
| Medicare | 20.5 | 86.1 |  | 7.4 | 87.9 |  |
| Other Public^4^ | 9.5 | 0 |  | 4.3 | 0 |  |
| Private or Other | 57.9 | 12.1 |  | 81.8 | 11.5 |  |
| Uninsured | 12.1 | 1.8 |  | 6.5 | 0.5 |  |
| **Self-Reported Comorbidities** |  |  |  |  |  |  |
| Hypertension | 61.5 | 53.1 | 0.171 | 40.9 | 46.1 | 0.105 |
| Hyperlipidemia | 45.3 | 43.5 | 0.037 | 39.2 | 39.4 | 0.006 |
| Diabetes | 21.9 | 19.7 | 0.054 | 12.6 | 10.3 | 0.074 |
| Myocardial Infarction | 7.7 | 4.8 | 0.119 | 3.1 | 2.7 | 0.021 |
| Stroke | 7.5 | 4.1 | 0.144 | 1.1 | 3.2 | 0.142 |
| COPD | 13.6 | 7 | 0.218 | 1.3 | 2.3 | 0.075 |
| Cancer | 17.2 | 15 | 0.058 | 21.9 | 19.6 | 0.057 |
| **US Region^5^** |  |  | 0.132 |  |  | 0.077 |
| Midwest | 22.5 | 21.3 |  | 19.5 | 19.2 |  |
| Northeast | 17.5 | 13.9 |  | 22.9 | 22.9 |  |
| South | 39 | 39.6 |  | 34.7 | 37.7 |  |
| West | 21 | 25.3 |  | 22.8 | 20.2 |  |

aSMD, absolute standardized mean difference. We compared the distribution of the covariates between the two populations using absolute standardized mean differences or, for discrete variables with multiple categories, Mahalanobis distances.

^1^ Low Educational Attainment was defined as less than a Bachelor’s degree
^2^ Higher Educational Attainment was defined as Bachelor’s degree or higher
^3^ National estimates based on survey weights for the 2019 National Health Interview Survey  ^4^ For adults age 66 years, individuals who were dually enrolled in Medicare and Medicaid are included in the “Medicare” category rather than the “other public” category, consistent with the approach used by NHIS. ^5^ US Census Bureau regions

**Table B. Association of Medicare Eligibility and Enrollment on Health Care Access, Prescription Medications, and Financial Strain** **After Adjusting for Self-Reported Comorbidities**^1^

|  |  | **Population Size^2^** | **Adults Age 64 years** | **Adults Age 66 years** | **Effect of Medicare Eligibility** | **P-Value** | **Effect of Medicare Enrollment** | **P-Value** |
| --- | --- | --- | --- | --- | --- | --- | --- | --- |
| **Health Care Access** | | | | | | | | |
| **Recent Physician Visit** | **Low-income** | 3,099,031 | 93.5% | 89.3% | -3.1%  (-8.7% to 2.5%) | 0.27 | -5.0%  (-14.0% to 3.9%) | 0.27 |
|  | **Higher-income** | 4,252,330 | 91.6% | 93.7% | 2.3%  (-2.4% to 7.1%) | 0.34 | 3.0%  (-3.1% to 9.0%) | 0.33 |
|  | **Overall** | 7,351,361 | 92.4% | 91.9% | 0.1%  (-3.5% to 3.7%) | 0.96 | 0.1%  (-4.9% to 5.2%) | 0.96 |
| **Delayed Medical Care Due to Cost** | **Low-income** | 3,097,139 | 14.7% | 6.2% | -7.4%  (-13.2% to -1.5%) | 0.01 | -11.9%  (-21.3% to -2.4%) | 0.014 |
|  | **Higher-income** | 4,252,330 | 3.5% | 2.4% | -1.3%  (-4.2% to 1.5%) | 0.36 | -1.7%  (-5.4% to 2.0%) | 0.36 |
|  | **Overall** | 7,349,469 | 8.3% | 3.9% | -3.8%  (-6.9% to -0.8%) | 0.02 | -5.4%  (-9.7% to -1.0%) | 0.02 |
| **Did Not Seek Medical Care Due to Cost** | **Low-income** | 3,097,139 | 14.3% | 6.0% | -7.0%  (-12.7% to -1.4%) | 0.014 | -11.3%  (-20.6% to -2.1%) | 0.02 |
|  | **Higher-income** | 4,252,330 | 3.5% | 0.6% | -3.0%  (-5.4% to -0.5%) | 0.02 | -3.8%  (-6.9% to -0.7%) | 0.02 |
|  | **Overall** | 7,349,469 | 8.2% | 2.8% | -4.6%  (-7.4% to -1.8%) | 0.001 | -6.4%  (-10.4% to -2.4%) | 0.002 |
| **Prescription Medications** | | | | | | | | |
| **Delayed Filling Prescription to Save Money^3^** | **Low-income** | 2,564,279 | 5.3% | 3.5% | -1.6%  (-5.4% to 2.2%) | 0.41 | -2.0%  (-6.8% to 2.8%) | 0.41 |
|  | **Higher-income** | 3,686,700 | 8.1% | 5.4% | -2.2%  (-6.1% to 1.7%) | 0.28 | -3.0%  (-8.5% to 2.4%) | 0.27 |
|  | **Overall** | 6,250,979 | 9.9% | 9.1% | -0.8%  (-8.2% to 6.5%) | 0.83 | -1.4%  (-13.6% to 10.9%) | 0.83 |
| **Needed Prescription Medication but Did Not Get it Due to Cost** | **Low-income** | 3,093,289 | 14.1% | 10.0% | -2.7%  (-9.5% to 4.1%) | 0.43 | -4.4%  (-15.3% to 6.6%) | 0.43 |
|  | **Higher-income** | 4,252,330 | 4.3% | 3.9% | -0.3%  (-3.7% to 3.1%) | 0.88 | -0.3%  (-4.7% to 4.0%) | 0.88 |
|  | **Overall** | 7,345,619 | 8.6% | 6.4% | -1.3%  (-4.9% to 2.4%) | 0.50 | -1.8%  (-6.9% to 3.3%) | 0.50 |
| **Skipped Medication Doses to Save Money^3^** | **Low-income** | 2,564,279 | 9.9% | 9.1% | -0.8%  (-8.2% to 6.5%) | 0.83 | -1.4%  (-13.6% to 10.9%) | 0.83 |
|  | **Higher-income** | 3,686,700 | 5.0% | 2.7% | -2.2%  (-6.0% to 1.6%) | 0.26 | -2.8%  (-7.6% to 2.0%) | 0.25 |
|  | **Overall** | 6,250,979 | 7.1% | 5.2% | -1.7%  (-5.5% to 2.2%) | 0.40 | -2.3%  (-7.8% to 3.1%) | 0.40 |
| **Took Less Medication to Save Money^3^** | **Low-income** | 2,564,279 | 14.2% | 9.1% | -5.0%  (-13.1% to 3.0%) | 0.22 | -8.5%  (-21.9% to 5.0%) | 0.22 |
|  | **Higher-income** | 3,686,700 | 2.9% | 2.3% | -0.8%  (-3.5% to 1.9%) | 0.57 | -1.0%  (-4.4% to 2.4%) | 0.57 |
|  | **Overall** | 6,250,979 | 7.7% | 5.0% | -2.5%  (-6.3% to 1.4%) | 0.21 | -3.5%  (-8.9% to 2.0%) | 0.21 |
| **Financial Strain Due to Medical Bills** | | | | | | | | |
| **Get Sick or Have Accident, Worry about Paying Medical Bills** | **Low-income** | 3,096,517 | 65.8% | 50.8% | -14.9%  (-24.8% to -5.0%) | 0.003 | -24.0%  (-40.2% to -7.8%) | 0.004 |
|  | **Higher-income** | 4,249,835 | 40.5% | 27.2% | -13.1%  (-21.5% to -4.7%) | 0.002 | -16.7%  (-27.5% to -5.9%) | 0.002 |
|  | **Overall** | 7,346,352 | 51.5% | 36.8% | -13.9%  (-20.8% to -7.0%) | <0.001 | -19.5%  (-29.3% to -9.7%) | <0.001 |
| **Problems Paying Medical Bills** | **Low-income** | 3,099,031 | 33.1% | 20.7% | -10.9%  (-20.2% to -1.5%) | 0.02 | -17.5%  (-32.6% to -2.5%) | 0.02 |
|  | **Higher-income** | 4,238,580 | 9.5% | 5.2% | -4.3%  (-8.7% to 0.2%) | 0.06 | -5.4%  (-11.0% to 0.3%) | 0.06 |
|  | **Overall** | 7,337,610 | 19.8% | 11.5% | -7.1%  (-12.2% to -2.0%) | 0.007 | -9.8%  (-17.0% to -2.7%) | 0.007 |
| **Unable to Pay Medical Bills^4^** | **Low-income** | 842,588 | 77.4% | 53.4% | -27.3%  (-46.4% to -8.2%) | 0.005 | -52.1%  (-91.8% to -12.5%) | 0.01 |
|  | **Higher-income** | 310,837 | 38.9% | 43.1% | 4.5%  (-29.9% to 38.9%) | 0.80 | 5.3%  (-34.7% to 45.2%) | 0.80 |
|  | **Overall** | 1,153,426 | 67.0% | 50.7% | -13.8%  (-31.4% to 3.8%) | 0.13 | -23.2%  (-52.9% to 6.6%) | 0.13 |

^1^ Adults with a missing response were excluded from the analysis of the respective outcome (<1% across all outcomes)

^2^ National estimates based on survey weights for the 2019 National Health Interview Survey

^3^ Among adults taking prescription medications

^4^ Among adults reporting problems paying medical bills

**Table C. Association of Medicare Eligibility and Enrollment on Health Care Access, Prescription Medications, and Financial Strain by Income Level after Adjusting for Employment**^1^

|  |  | **Population Size^2^** | **Adults Age 64 years** | **Adults Age 66 years** | **Effect of Medicare Eligibility** | **P-Value** | **Effect of Medicare Enrollment** | **P-Value** |
| --- | --- | --- | --- | --- | --- | --- | --- | --- |
| **Health Care Access** | | | | | | | | |
| **Recent Physician Visit** | **Low-income** | 3,034,609 | 93.4% | 88.9% | -5.1%  (-11.6%, 1.4%) | 0.12 | -8.6%  (-19.6%, 2.4%) | 0.12 |
|  | **Higher-income** | 4,151,697 | 92.1% | 93.6% | 1.5%  (-3.3%, 6.3%) | 0.54 | 1.9%  (-4.2%, 8.0%) | 0.54 |
|  | **Overall** | 7,186,306 | 92.7% | 91.7% | -1.5%  (-5.5%, 2.5%) | 0.47 | -2.1%  (-7.9%, 3.6%) | 0.47 |
| **Delayed Medical Care Due to Cost** | **Low-income** | 3,034,609 | 14.8% | 6.2% | -7.5%  (-13.8%, -1.2%) | 0.02 | -12.6%  (-23.4%, -1.9%) | 0.02 |
|  | **Higher-income** | 4,153,747 | 2.9% | 2.4% | -0.4%  (-3.0%, 2.1%) | 0.75 | -0.5%  (-3.8%, 2.7%) | 0.75 |
|  | **Overall** | 7,188,356 | 8.1% | 4.0% | -3.4%  (-6.6%, -0.3%) | 0.03 | -4.9%  (-9.5%, -0.4%) | 0.03 |
| **Did Not Seek Medical Care Due to Cost** | **Low-income** | 3,034,609 | 15.2% | 5.9% | -7.9%  (-13.9%, -1.9%) | 0.01 | -13.3%  (-23.6%, -3.0%) | 0.01 |
|  | **Higher-income** | 4,153,747 | 2.9% | 0.6% | -1.9%  (-4.0%, 0.1%) | 0.06 | -2.5%  (-5.1%, 0.1%) | 0.06 |
|  | **Overall** | 7,188,356 | 8.3% | 2.8% | -4.5%  (-7.3%, -1.7%) | 0.002 | -6.4%  (-10.6%, -2.3%) | 0.002 |
| **Prescription Medications** | | | | | | | | |
| **Delayed Filling Prescription to Save Money**^3^ | **Low-income** | 2,518,633 | 11.8% | 8.6% | -2.4%  (-9.9%, 5.1%) | 0.53 | -4.1%  (-17.1%, 8.9%) | 0.40 |
|  | **Higher-income** | 3,600,972 | 4.6% | 3.6% | -1.0%  (-4.7%, 2.7%) | 0.59 | -1.3%  (-5.9%, 3.4%) | 0.59 |
|  | **Overall** | 6,119,605 | 7.7% | 5.6% | -1.5%  (-5.3%, 2.3%) | 0.44 | -2.1%  (-7.5%, 3.3%) | 0.44 |
| **Needed Prescription Medication but Did Not Get it Due to Cost** | **Low-income** | 3,034,609 | 14.8% | 9.5% | -3.4%  (-10.4%, 3.6%) | 0.34 | -5.8%  (-17.7%, 6.2%) | 0.35 |
|  | **Higher-income** | 4,153,747 | 3.7% | 4.0% | 0.5%  (-2.4%, 3.3%) | 0.75 | 0.6%  (-3.1%, 4.3%) | 0.75 |
|  | **Overall** | 7,188,356 | 8.6% | 6.2% | -1.2%  (-4.8%, 2.4%) | 0.51 | -1.7%  (-6.9%, 3.4%) | 0.51 |
| **Skipped Medication Doses to Save Money**^3^ | **Low-income** | 2,518,633 | 9.3% | 9.6% | 0.8%  (-6.9%, 8.5%) | 0.83 | 1.4%  (-11.8%, 14.7%) | 0.83 |
|  | **Higher-income** | 3,600,972 | 4.3% | 2.7% | -1.7%  (-5.3%, 1.8%) | 0.34 | -2.2%  (-6.7%, 2.3%) | 0.34 |
|  | **Overall** | 6,119,605 | 6.5% | 5.4% | -0.6%  (-4.4%, 3.2%) | 0.76 | -0.8%  (-6.3%, 4.6%) | 0.76 |
| **Took Less Medication to Save Money**^3^ | **Low-income** | 2,518,633 | 13.6% | 9.5% | -3.7%  (-12.1%, 4.8%) | 0.39 | -6.3%  (-20.9%, 8.3%) | 0.40 |
|  | **Higher-income** | 3,600,972 | 2.2% | 2.3% | -0.1%  (-2.5%, 2.2%) | 0.92 | -0.2%  (-3.2%, 2.8%) | 0.92 |
|  | **Overall** | 6,119,605 | 7.1% | 5.2% | -1.6%  (-5.6%, 2.4%) | 0.43 | -2.3%  (-8.1%, 3.4%) | 0.43 |
| **Financial Strain Due to Medical Bills** | | | | | | | | |
| **Get Sick or Have Accident, Worry about Paying Medical Bills** | **Low-income** | 3,032,095 | 66.7% | 51.8% | -14.0%  (-24.3%, -3.7%) | 0.008 | -23.6%  (-41.2%, -6.0%) | 0.009 |
|  | **Higher-income** | 4,151,252 | 39.7% | 27.2% | -10.6%  (-19.2%, -2.1%) | 0.01 | -13.6%  (-24.6%, -2.6%) | 0.02 |
|  | **Overall** | 7,183,348 | 51.6% | 37.2% | -13.0%  (-20.0%, -6.0%) | <0.001 | -18.6%  (-28.8%, -8.5%) | <0.001 |
| **Problems Paying Medical Bills** | **Low-income** | 3,034,609 | 33.3% | 20.4% | -10.1%  (-19.8%, -0.5%) | 0.04 | -17.1%  (-33.5%, -0.7%) | 0.04 |
|  | **Higher-income** | 4,137,947 | 9.1% | 5.3% | -3.0%  (-7.7%, 1.8%) | 0.22 | -3.8%  (-9.9%, 2.3%) | 0.22 |
|  | **Overall** | 7,172,555 | 19.8% | 11.4% | -6.4%  (-11.6%, -1.2%) | 0.02 | -9.2%  (-16.6%, -1.7%) | 0.02 |
| **Unable to Pay Medical Bills^4^** | **Low-income** | 827,759 | 76.7% | 55.1% | -20.2%  (-41.5%, 1.0%) | 0.06 | -37.8%  (-79.3%, 3.6%) | 0.07 |
|  | **Higher-income** | 296,833 | 34.3% | 43.1% | 1.9%  (-31.7%, 35.5%) | 0.91 | 2.3%  (-37.6%, 42.1%) | 0.91 |
|  | **Overall** | 1,124,592 | 65.8% | 51.8% | -12.0%  (-30.9%, 6.8%) | 0.21 | -19.7%  (-50.6%, 11.1%) | 0.21 |

The relationship between Medicare eligibility, enrollment, and employment is likely to be complex and under certain causal structures (e.g., some structures in which the decision to maintain employment is affected by Medicare eligibility or enrollment) adjustment for employment using standard regression methods may be inappropriate. Hence, analyses adjusted for employment are offered only as exploratory stability analyses.

^1^ Adults with a missing response were excluded from the analysis of the respective outcome (<1% across all outcomes)

^2^ National estimates based on survey weights for the 2019 National Health Interview Survey

^3^ Among adults taking prescription medications

^4^ Among adults reporting problems paying medical bills

**Table D. Association of Medicare Eligibility and Enrollment on Health Care Access, Prescription Medications, and Financial Strain After Adjusting for Self-Reported Comorbidities, Employment, and other Baseline Characteristics**^1,2^

|  |  | **Population Size^3^** | **Adults Age 64 years** | **Adults Age 66 years** | **Effect of Medicare Eligibility** | **P-Value** | **Effect of Medicare Enrollment** | **P-Value** |
| --- | --- | --- | --- | --- | --- | --- | --- | --- |
| **Health Care Access** | | | | | | | | |
| **Recent Physician Visit** | **Low-income** | 2,946,918 | 93.5% | 89.4% | -2.8%  (-8.8% to 3.2%) | 0.36 | -4.6%  (-14.4% to 5.2%) | 0.36 |
|  | **Higher-income** | 4,111,995 | 92.0% | 93.5% | 2.2%  (-2.5% to 7.0%) | 0.36 | 2.8%  (-3.2% to 8.9%) | 0.36 |
|  | **Overall** | 7,058,912 | 92.7% | 91.9% | -0.2%  (-4.1% to 3.7%) | 0.93 | -0.3%  (-5.7% to 5.2%) | 0.93 |
| **Delayed Medical Care Due to Cost** | **Low-income** | 2,946,918 | 14.8% | 6.3% | -6.0%  (-12.0% to 0.1%) | 0.05 | -9.8%  (-19.8% to 0.1%) | 0.05 |
|  | **Higher-income** | 4,111,995 | 2.9% | 2.4% | -0.5%  (-3.0% to 1.9%) | 0.67 | -0.7%  (-3.9% to 2.5%) | 0.67 |
|  | **Overall** | 7,058,912 | 8.0% | 4.0% | -2.8% (-5.9% to 0.3%) | 0.08 | -3.9%  (-8.2% to 0.5%) | 0.08 |
| **Did Not Seek Medical Care Due to Cost** | **Low-income** | 2,946,918 | 14.0% | 6.0% | -5.6%  (-11.1% to -0.1%) | 0.05 | -9.2%  (-18.3% to 0.0%) | 0.05 |
|  | **Higher-income** | 4,111,995 | 3.0% | 0.6% | -1.9%  (-4.0% to 0.1%) | 0.07 | -2.5%  (-5.1% to 0.2%) | 0.07 |
|  | **Overall** | 7,058,912 | 7.7% | 2.8% | -3.4%  (-6.1% to -0.7%) | 0.01 | -4.8%  (-8.6% to -1.0%) | 0.01 |
| **Prescription Medications** | | | | | | | | |
| **Delayed Filling Prescription to Save Money**^4^ | **Low-income** | 2,450,569 | 9.5% | 8.6% | 0.8%  (-5.5% to 7.1%) | 0.81 | 1.3%  (-9.3% to 11.9%) | 0.81 |
|  | **Higher-income** | 3,561,270 | 4.7% | 3.6% | -0.5%  (-3.7% to 2.6%) | 0.74 | -0.7%  (-4.6% to 3.3%) | 0.74 |
|  | **Overall** | 6,011,839 | 6.7% | 5.6% | 0.2%  (-3.2% to 3.5%) | 0.92 | 0.2%  (-4.5% to 4.9%) | 0.92 |
| **Needed Prescription Medication but Did Not Get it Due to Cost** | **Low-income** | 2,946,918 | 12.0% | 9.6% | 1.1%  (-4.9% to 7.0%) | 0.72 | 1.8%  (-8.0% to 11.5%) | 0.72 |
|  | **Higher-income** | 4,111,995 | 3.8% | 4.1% | 0.6%  (-2.3% to 3.6%) | 0.67 | 0.8%  (-2.9% to 4.6%) | 0.67 |
|  | **Overall** | 7,058,912 | 7.3% | 6.3% | 1.0%  (-2.2% to 4.1%) | 0.55 | 1.3%  (-3.1% to 5.8%) | 0.55 |
| **Skipped Medication Doses to Save Money**^4^ | **Low-income** | 2,450,569 | 7.4% | 9.6% | 2.3%  (-3.9% to 8.4%) | 0.47 | 3.8%  (-6.6% to 14.2%) | 0.40 |
|  | **Higher-income** | 3,561,270 | 4.4% | 2.7% | -1.2%  (-4.2% to 1.8%) | 0.43 | -1.5%  (-5.3% to 2.3%) | 0.43 |
|  | **Overall** | 6,011,839 | 5.7% | 5.5% | 0.3%  (-2.9% to 3.5%) | 0.85 | 0.4%  (-4.1% to 4.9%) | 0.85 |
| **Took Less Medication to Save Money**^4^ | **Low-income** | 2,450,569 | 11.9% | 9.6% | -1.8%  (-8.6% to 5.1%) | 0.61 | -3.0%  (-14.5% to 8.5%) | 0.61 |
|  | **Higher-income** | 3,561,270 | 2.3% | 2.3% | -0.1%  (-2.5% to 2.3%) | 0.95 | -0.1%  (-3.1% to 2.9%) | 0.95 |
|  | **Overall** | 6,011,839 | 6.3% | 5.2% | -0.8%  (-4.1% to 2.6%) | 0.65 | -1.1%  (-5.8% to 3.6%) | 0.65 |
| **Financial Strain Due to Medical Bills** | | | | | | | | |
| **Get Sick or Have Accident, Worry about Paying Medical Bills** | **Low-income** | 2,944,404 | 65.2% | 51.2% | -13.3%  (-23.3% to -3.4%) | 0.008 | -21.9%  (-38.5% to -5.3%) | 0.01 |
|  | **Higher-income** | 4,109,500 | 40.1% | 26.8% | -11.2%  (-19.6% to -2.8%) | 0.009 | -14.3%  (-25.0% to -3.5%) | 0.009 |
|  | **Overall** | 7,053,904 | 50.9% | 36.6% | -12.0%  (-18.6% to -5.4%) | <0.001 | -16.8%  (-26.2% to -7.5%) | <0.001 |
| **Problems Paying Medical Bills** | **Low-income** | 2,946,918 | 31.3% | 20.7% | -4.2%  (-12.9% to 4.5%) | 0.34 | -6.9%  (-21.1% to 7.3%) | 0.34 |
|  | **Higher-income** | 4,098,245 | 9.2% | 5.4% | -2.8%  (-7.4% to 1.9%) | 0.25 | -3.5%  (-9.4% to 2.5%) | 0.25 |
|  | **Overall** | 7,045,162 | 18.8% | 11.5% | -3.4%  (-8.1% to 1.2%) | 0.14 | -4.8%  (-11.3% to 1.7%) | 0.15 |
| **Unable to Pay Medical Bills^5^** | **Low-income** | 773,827 | 75.2% | 55.1% | -16.4%  (-33.0% to 0.2%) | 0.05 | -32.9%  (-68.5% to 2.7%) | 0.07 |
|  | **Higher-income** | 296,833 | 34.3% | 43.1% | 1.6%  (-25.8% to 29.0%) | 0.91 | 1.9%  (-30.7% to 34.5%) | 0.91 |
|  | **Overall** | 1,070,660 | 63.9% | 51.8% | -3.9%  (-19.4% to 11.6%) | 0.62 | -6.7%  (-33.2% to 19.8%) | 0.62 |

^1^ Adults with a missing response were excluded from the analysis of the respective outcome (<1% across all outcomes)

^2^ Other baseline characteristics include: gender, race/ethnicity, marriage status, education, and US region

^3^ National estimates based on survey weights for the 2019 National Health Interview Survey

^4^ Among adults taking prescription medications

^5^ Among adults reporting problems paying medical bills

**Table E. Association of Medicare Eligibility and Enrollment on Health Care Access, Prescription Medications, and Financial Strain by Income Level Using: Alternative Definition for Low-Income Population**^1^ **(≤200% FPL)**

|  |  | **Population Size^2^** | **Adults Age 64 years** | **Adults Age 66 years** | **Effect of Medicare Eligibility** | **P-Value** | **Effect of Medicare Enrollment** | **P-Value** |
| --- | --- | --- | --- | --- | --- | --- | --- | --- |
| **Health Care Access** | | | | | | | | |
| **Recent Physician Visit** | **Low-income** | 2,026,930 | 93.0% | 85.3% | -7.7%  (-16.0% to 0.6%) | 0.07 | -12.9%  (-26.7% to 1.0%) | 0.07 |
|  | **Higher-income** | 5,388,473 | 92.3% | 94.4% | 2.1%  (-1.9% to 6.1%) | 0.30 | 2.8%  (-2.5% to 8.2%) | 0.30 |
|  | **Overall** | 7,415,402 | 92.5% | 92.0% | -0.5%  (-4.2% to 3.2%) | 0.80 | -0.8%  (-6.0% to 4.5%) | 0.80 |
| **Delayed Medical Care Due to Cost** | **Low-income** | 2,025,038 | 14.6% | 7.1% | -7.5%  (-15.8% to 0.7%) | 0.07 | -12.7%  (-26.9% to 1.5%) | 0.08 |
|  | **Higher-income** | 5,390,523 | 6.0% | 2.7% | -3.3%  (-6.2% to -0.3%) | 0.03 | -4.4%  (-8.3% to -0.4%) | 0.03 |
|  | **Overall** | 7,415,561 | 8.4% | 3.9% | -4.5%  (-7.7% to -1.3%) | 0.005 | -6.4%  (-11.0% to -1.8%) | 0.006 |
| **Did Not Seek Medical Care Due to Cost** | **Low-income** | 2,025,038 | 16.8% | 6.7% | -10.2%  (-18.8% to -1.5%) | 0.02 | -17.1%  (-32.4% to -1.8%) | 0.03 |
|  | **Higher-income** | 5,390,523 | 5.7% | 1.4% | -4.3%  (-6.9% to -1.7%) | 0.001 | -5.8%  (-9.4% to -2.2%) | 0.001 |
|  | **Overall** | 7,415,561 | 8.8% | 2.8% | -6.0%  (-9.1% to -2.9%) | <0.001 | -8.6%  (-13.0% to -4.1%) | <0.001 |
| **Prescription Medications** | | | | | | | | |
| **Delayed Filling Prescription to Save Money**^3^ | **Low-income** | 1,650,196 | 12.3% | 9.2% | -3.1%  (-12.9% to 6.6%) | 0.53 | -5.6%  (-23.0% to 11.8%) | 0.53 |
|  | **Higher-income** | 4,660,274 | 6.6% | 4.1% | -2.6%  (-6.2% to 1.1%) | 0.17 | -3.5%  (-8.4% to 1.4%) | 0.17 |
|  | **Overall** | 6,310,471 | 8.2% | 5.3% | -2.8%  (-6.6% to 0.9%) | 0.14 | -4.1%  (-9.5% to 1.3%) | 0.14 |
| **Needed Prescription Medication but Did Not Get it Due to Cost** | **Low-income** | 2,025,038 | 18.4% | 10.3% | -8.1%  (-17.9% to 1.8%) | 0.11 | -13.6%  (-30.7% to 3.5%) | 0.12 |
|  | **Higher-income** | 5,386,673 | 5.3% | 4.9% | -0.4%  (-3.8% to 3.0%) | 0.82 | -0.5%  (-5.1% to 4.1%) | 0.82 |
|  | **Overall** | 7,411,711 | 9.0% | 6.3% | -2.6%  (-6.4% to 1.2%) | 0.18 | -3.7%  (-9.2% to 1.7%) | 0.18 |
| **Skipped Medication Doses to Save Money**^3^ | **Low-income** | 1,650,196 | 9.6% | 10.4% | 0.8%  (-9.5% to 11.1%) | 0.88 | 1.4%  (-17.0% to 19.9%) | 0.88 |
|  | **Higher-income** | 4,660,274 | 6.0% | 3.5% | -2.5%  (-6.0% to 0.9%) | 0.15 | -3.4%  (-8.0% to 1.2%) | 0.15 |
|  | **Overall** | 6,310,471 | 7.0% | 5.2% | -1.8%  (-5.6% to 2.0%) | 0.36 | -2.6%  (-8.0% to 2.9%) | 0.36 |
| **Took Less Medication to Save Money**^3^ | **Low-income** | 1,650,196 | 15.4% | 11.1% | -4.3%  (-15.8% to 7.2%) | 0.46 | -7.7%  (-28.2% to 12.9%) | 0.47 |
|  | **Higher-income** | 4,660,274 | 4.7% | 2.9% | -1.8%  (-4.7% to 1.1%) | 0.23 | -2.4%  (-6.3% to 1.5%) | 0.23 |
|  | **Overall** | 6,310,471 | 7.6% | 5.0% | -2.6%  (-6.4% to 1.2%) | 0.17 | -3.8%  (-9.3% to 1.7%) | 0.17 |
| **Financial Strain Due to Medical Bills** | | | | | | | | |
| **Get Sick or Have Accident, Worry about Paying Medical Bills** | **Low-income** | 2,024,416 | 65.4% | 54.4% | -11.0%  (-23.7% to 1.8%) | 0.09 | -18.4%  (-39.9% to 3.1%) | 0.09 |
|  | **Higher-income** | 5,388,028 | 46.8% | 30.9% | -15.9%  (-23.8% to -8.0%) | <0.001 | -21.4%  (-32.4% to -10.5%) | <0.001 |
|  | **Overall** | 7,412,444 | 51.9% | 37.2% | -14.8%  (-21.8% to -7.8%) | <0.001 | -21.1%  (-31.4% to -10.9%) | <0.001 |
| **Problems Paying Medical Bills** | **Low-income** | 2,026,930 | 37.9% | 22.0% | -15.9%  (-28.1% to -3.6%) | 0.01 | -26.7%  (-47.6% to -5.8%) | 0.01 |
|  | **Higher-income** | 5,374,723 | 13.4% | 7.6% | -5.8%  (-10.6% to -1.0%) | 0.02 | -7.8%  (-14.4% to -1.3%) | 0.02 |
|  | **Overall** | 7,401,652 | 20.3% | 11.4% | -8.8%  (-14.1% to -3.5%) | 0.001 | -12.6%  (-20.2% to -4.9%) | 0.001 |
| **Unable to Pay Medical Bills^4^** | **Low-income** | 612,512 | 78.9% | 51.9% | -27.0%  (-52.2% to -1.7%) | 0.04 | -47.6%  (-90.4% to -4.7%) | 0.030 |
|  | **Higher-income** | 564,501 | 54.4% | 49.3% | -5.1%  (-30.4% to 20.3%) | 0.69 | -7.9%  (-48.0% to 32.2%) | 0.70 |
|  | **Overall** | 1,177,013 | 67.2% | 50.7% | -16.6%  (-35.1% to 2.0%) | 0.08 | -27.4%  (-58.1% to 3.3%) | 0.08 |

^1^ Adults with a missing response were excluded from the analysis of the respective outcome (<1% across all outcomes)

^2^ National estimates based on survey weights for the 2019 National Health Interview Survey

^3^ Among adults taking prescription medications
^4^ Among adults reporting problems paying medical bills

**Table F: Baseline Characteristics of the U.S. Population Ages 63-64 and 66-67 Years by Income Level**^1^

|  | **Low-Income**^2^ | | | **Higher-Income**^3^ | | | **Overall** | | |
| --- | --- | --- | --- | --- | --- | --- | --- | --- | --- |
|  | **Age 64 years** | **Age 66 years** | **P-Value** | **Age 64 years** | **Age 66 years** | **P-Value** | **Age 64 years** | **Age 66 years** | **P-Value** |
| **Population Size** | 3,463,383 | 2,735,508 |  | 4,781,272 | 3,916,014 |  | 8,244,655 | 6,651,522 |  |
| **Mean Age** | 64 | 66 |  | 64 | 66 |  | 64 | 66 |  |
| **Female (%)** | 57.4 | 60.0 | 0.49 | 49.6 | 52.6 | 0.32 | 52.9 | 55.6 | 0.24 |
| **Race (%)** |  |  | 0.02 |  |  | 0.43 |  |  | 0.09 |
| White | 55.4 | 65.9 |  | 80.2 | 82.7 |  | 69.8 | 75.8 |  |
| Black | 18.8 | 12 |  | 5.2 | 6.2 |  | 10.9 | 8.6 |  |
| Hispanic | 17.9 | 16.8 |  | 7.7 | 4.8 |  | 12 | 9.7 |  |
| Asian | 4.2 | 4.2 |  | 5.8 | 5.1 |  | 5.1 | 4.7 |  |
| Other | 3.6 | 1.1 |  | 1.1 | 1.2 |  | 2.1 | 1.2 |  |
| **Married** | 48.0 | 47.5 | 0.91 | 80.6 | 78.2 | 0.27 | 67.0 | 65.6 | 0.55 |
| **Education (Bachelors Degree or Higher)** | 13.1 | 13.7 | 0.79 | 39.1 | 43.7 | 0.147 | 28.2 | 31.4 | 0.14 |
| **Employed** | 36.5 | 22.1 | <0.001 | 64 | 43 | <0.001 | 52.5 | 34.4 | <0.001 |
| **Insurance (%)** |  |  | <0.001 |  |  | <0.001 |  |  | <0.001 |
| Medicare | 25.6 | 90.6 |  | 5.9 | 89.4 |  | 14.2 | 89.9 |  |
| Other Public | 15.9 | 0 |  | 0.8 | 0 |  | 7.2 | 0 |  |
| Private or Other | 43.3 | 7.4 |  | 87.0 | 10.2 |  | 68.7 | 9.0 |  |
| Uninsured | 15.3 | 2.0 |  | 6.2 | 0.4 |  | 10 | 1.0 |  |
| **Self-Reported Comorbidities** |  |  |  |  |  |  |  |  |  |
| Hypertension | 62.7 | 63.7 | 0.79 | 46.9 | 47.7 | 0.80 | 53.5 | 54.3 | 0.76 |
| Hyperlipidemia | 44.8 | 49.6 | 0.22 | 41.2 | 42.7 | 0.64 | 42.7 | 45.5 | 0.26 |
| Diabetes | 26.3 | 25.1 | 0.70 | 14.0 | 13.3 | 0.73 | 19.2 | 18.1 | 0.56 |
| Myocardial Infarction | 9.4 | 8.2 | 0.54 | 4.5 | 2.7 | 0.098 | 6.6 | 4.9 | 0.12 |
| Stroke | 8.5 | 7.3 | 0.54 | 2.9 | 2.0 | 0.34 | 5.2 | 4.2 | 0.29 |
| COPD | 15.2 | 10.3 | 0.04 | 5.2 | 3.2 | 0.01 | 9.4 | 6.2 | 0.008 |
| Cancer | 12.3 | 17.4 | 0.05 | 17.8 | 17.6 | 0.95 | 15.5 | 17.5 | 0.23 |
| **US Region**^4^ |  |  | 0.45 |  |  | 0.33 |  |  | 0.52 |
| Midwest | 20.8 | 18.8 |  | 23.0 | 23.4 |  | 22.1 | 21.5 |  |
| Northeast | 15.9 | 12.9 |  | 20.6 | 20.3 |  | 18.6 | 17.3 |  |
| South | 41.5 | 43.4 |  | 30.3 | 34.6 |  | 35.0 | 38.2 |  |
| West | 21.7 | 24.9 |  | 26.1 | 21.7 |  | 24.3 | 23.0 |  |

National estimates based on survey weights for the 2019 National Health Interview Survey

^1^ Adults missing information on age, Medicare enrollment, and income were excluded (<0.5%)

^2^ Low-income was defined as family income ≤300% of the federal poverty line.

^3^ Higher-income was defined as family income >300% federal poverty line.

^4^ US Census Bureau regions

**Table G. Association of Medicare Eligibility and Enrollment on Health Care Access, Prescription Medications, and Financial Strain by Income Level Using Wider Age Ranges**^1^ **(63-64 years vs. 66-67 years)**

|  |  | **Adults Age 64 years** | **Adults Age 66 years** | **Effect of Medicare Eligibility** | **P-Value** | **Effect of Medicare Enrollment** | **P-Value** |
| --- | --- | --- | --- | --- | --- | --- | --- |
| **Health Care Access** | | | | | | | |
| **Recent Physician Visit** | **Low-income** | 91.0% | 91.2% | 0.2%  (-4.4% to 4.8%) | 0.94 | 0.3%  (-6.8% to 7.3%) | 0.94 |
|  | **Higher-income** | 91.3% | 94.5% | 3.3%  (-0.0% to 6.6%) | 0.05 | 3.9%  (0.0% to 7.8%) | 0.05 |
|  | **Overall** | 91.2% | 93.2% | 2.0%  (-0.7% to 4.7%) | 0.140 | 2.6%  (-0.9% to 6.1%) | 0.14 |
| **Delayed Medical Care Due to Cost** | **Low-income** | 17.7% | 6.9% | -10.8%  (-15.8% to -5.7%) | <0.001 | -16.5%  (-24.4% to -8.7%) | <0.001 |
|  | **Higher-income** | 4.9% | 2.0% | -2.9%  (-5.2% to -0.6%) | 0.01 | -3.5%  (-6.2% to -0.7%) | 0.02 |
|  | **Overall** | 10.3% | 4.0% | -6.2%  (-8.8% to -3.7%) | <0.001 | -8.2%  (-11.6% to -4.8%) | <0.001 |
| **Did Not Seek Medical Care Due to Cost** | **Low-income** | 18.0% | 6.9% | -11.2%  (-16.4% to -6.0%) | <0.001 | -17.2%  (-25.2% to -9.1%) | <0.001 |
|  | **Higher-income** | 3.8% | 1.2% | -2.6%  (-4.6% to -0.7%) | 0.008 | -3.2%  (-5.5% to -0.8%) | 0.008 |
|  | **Overall** | 9.8% | 3.5% | -6.3%  (-8.8% to -3.8%) | <0.001 | -8.3%  (-11.6% to -4.9%) | <0.001 |
| **Prescription Medications** | | | | | | | |
| **Delayed Filling Prescription to Save Money**^3^ | **Low-income** | 13.0% | 8.1% | -4.9%  (-10.0% to 0.2%) | 0.06 | -7.7%  (-15.7% to 0.4%) | 0.06 |
|  | **Higher-income** | 4.2% | 2.6% | -1.6%  (-4.1% to 0.9%) | 0.21 | -1.9%  (-5.0% to 1.1%) | 0.21 |
|  | **Overall** | 7.8% | 4.9% | -3.0%  (-5.5% to -0.4%) | 0.02 | -3.9%  (-7.4% to -0.5%) | 0.03 |
| **Needed Prescription Medication but Did Not Get it Due to Cost** | **Low-income** | 15.9% | 9.3% | -6.6%  (-11.8% to -1.5%) | 01 | -10.2%  (-18.2% to -2.1%) | .01 |
|  | **Higher-income** | 4.0% | 3.1% | -0.9%  (-3.2% to 1.4%) | 0.46 | -1.0%  (-3.8% to 1.7%) | 0.46 |
|  | **Overall** | 9.0% | 5.7% | -3.3%  (-6.0% to -0.7%) | 0.01 | -4.4%  (-7.9% to -0.9%) | 0.01 |
| **Skipped Medication Doses to Save Money**^3^ | **Low-income** | 9.4% | 8.4% | -1.0%  (-6.0% to 4.0%) | 0.69 | -1.6%  (-9.4% to 6.2%) | 0.69 |
|  | **Higher-income** | 3.6% | 2.4% | -1.3%  (-3.8% to 1.2%) | 0.30 | -1.6%  (-4.5% to 1.4%) | 0.30 |
|  | **Overall** | 6.0% | 4.9% | -1.2%  (-3.7% to 1.3%) | 0.35 | -1.6%  (-4.9% to 1.7%) | 0.35 |
| **Took Less Medication to Save Money**^3^ | **Low-income** | 11.6% | 8.3% | -3.3%  (-8.5% to 1.9%) | 0.21 | -5.2%  (-13.4% to 3.0%) | 0.21 |
|  | **Higher-income** | 3.2% | 1.6% | -1.6%  (-3.4% to 0.3%) | 0.10 | -1.9%  (-4.1% to 0.4%) | 0.10 |
|  | **Overall** | 6.7% | 4.4% | -2.3%  (-4.8% to 0.2%) | 0.07 | -3.0%  (-6.3% to 0.2%) | 0.07 |
| **Financial Strain Due to Medical Bills** | | | | | | | |
| **Get Sick or Have Accident, Worry about Paying Medical Bills** | **Low-income** | 63.7% | 49.4% | -14.2%  (-21.4% to -7.0%) | <0.001 | -21.8%  (-32.8% to -10.7%) | <0.001 |
|  | **Higher-income** | 40.8% | 27.6% | -13.2%  (-19.3% to -7.1%) | <0.001 | -15.8%  (-23.2% to -8.5%) | <0.001 |
|  | **Overall** | 50.4% | 36.6% | -13.8%  (-18.6% to -9.1%) | <0.001 | -18.3%  (-24.6% to -11.9%) | <0.001 |
| **Problems Paying Medical Bills** | **Low-income** | 29.7% | 18.3% | -11.4%  (-17.8% to -5.0%) | <0.001 | -17.5%  (-27.5% to -7.5%) | <0.001 |
|  | **Higher-income** | 7.5% | 5.3% | -2.2%  (-5.1% to 0.7%) | 0.14 | -2.6%  (-6.1% to 0.9%) | 0.14 |
|  | **Overall** | 16.8% | 10.6% | -6.2%  (-9.6% to -2.8%) | <0.001 | -8.1%  (-12.6% to -3.6%) | <0.001 |
| **Unable to Pay Medical Bills^4^** | **Low-income** | 77.6% | 58.0% | -19.6%  (-35.1% to -4.1%) | 0.01 | -34.5%  (-62.4% to -6.7%) | 0.02 |
|  | **Higher-income** | 37.4% | 47.1% | 9.7%  (-14.6% to 34.1%) | 0.43 | 12.0%  (-17.7% to 41.7%) | 0.43 |
|  | **Overall** | 67.2% | 54.9% | -1.4%  (-25.8% to 1.1%) | 0.07 | -19.6%  (-41.1% to 1.8%) | 0.07 |

^1^ Adults with a missing response were excluded from the analysis of the respective outcome (<1% across all outcomes)

^2^ National estimates based on survey weights for the 2019 National Health Interview Survey

^3^ Among adults taking prescription medications

^4^ Among adults reporting problems paying medical bills

**Table H. Association of Medicare Eligibility and Enrollment on Health Care Access, Prescription Medications, and Financial Strain by Education Level After Adjusting for Employment^1^**

|  |  | **Population Size^2^** | **Adults Age 64 years** | **Adults Age 66 years** | **Effect of Medicare Eligibility** | **P-Value** | **Effect of Medicare Enrollment** | **P-Value** |
| --- | --- | --- | --- | --- | --- | --- | --- | --- |
| **Health Care Access** | | | | | | | | |
| **Recent Physician Visit** | **Low Education** | 5074787 | 93.3% | 91.5% | -2.6%  (-7.6% to 2.5%) | 0.316 | -4.0%  (-11.9% to 3.9%) | 0.318 |
|  | **Higher Education** | 2048167 | 91.3% | 92.8% | 2.0%  (-4.0% to 8.0%) | 0.516 | 2.4%  (-4.8% to 9.5%) | 0.516 |
|  | **Overall** | 7122954 | 92.7% | 91.9% | -1.2%  (-5.3% to 2.8%) | 0.548 | -1.8%  (-7.6% to 4.0%) | 0.548 |
| **Delayed Medical Care Due to Cost** | **Low Education** | 5074787 | 9.5% | 5.3% | -3.4%  (-7.7% to 0.8%) | 0.109 | -5.4%  (-12.0% to 1.3%) | 0.112 |
|  | **Higher Education** | 2050217 | 4.6% | 0.9% | -3.4%  (-6.5% to -0.3%) | 0.030 | -4.0%  (-7.7% to -0.4%) | 0.031 |
|  | **Overall** | 7125005 | 8.1% | 4.0% | -3.3%  (-6.5% to -0.2%) | 0.039 | -4.8%  (-9.3% to -0.2%) | 0.040 |
| **Did Not Seek Medical Care Due to Cost** | **Low Education** | 5074787 | 10.2% | 3.9% | -5.1%  (-9.0% to -1.2%) | 0.010 | -8.0%  (-14.2% to -1.8%) | 0.012 |
|  | **Higher Education** | 2050217 | 3.4% | 0.3% | -2.9%  (-5.4% to -0.4%) | 0.025 | -3.4%  (-6.4% to -0.4%) | 0.025 |
|  | **Overall** | 7125005 | 8.3% | 2.8% | -4.4%  (-7.2% to -1.5%) | 0.003 | -6.3%  (-10.4% to -2.1%) | 0.003 |
| **Prescription Medications** | | | | | | | | |
| **Delayed Filling Prescription to Save Money**^3^ | **Low Education** | 4348280 | 6.7% | 5.8% | -0.3%  (-4.4% to 3.8%) | 0.882 | -0.5%  (-6.9% to 6.0%) | 0.882 |
|  | **Higher Education** | 1723051 | 7.2% | 4.9% | -1.7%  (-7.4% to 4.1%) | 0.566 | -2.0%  (-8.7% to 4.8%) | 0.567 |
|  | **Overall** | 6071331 | 6.8% | 5.6% | -0.7%  (-4.1% to 2.7%) | 0.705 | -0.9%  (-5.8% to 3.9%) | 0.705 |
| **Needed Prescription Medication but Did Not Get it Due to Cost** | **Low Education** | 7119996 | 8.4% | 6.7% | -1.0%  (-5.1% to 3.1%) | 0.632 | -1.6%  (-8.0% to 4.9%) | 0.634 |
|  | **Higher Education** | 5074787 | 6.0% | 5.3% | 1.3%  (-3.5% to 6.2%) | 0.590 | 1.6%  (-4.2% to 7.4%) | 0.589 |
|  | **Overall** | 2050217 | 7.7% | 6.3% | -0.3%  (-3.6% to 3.0%) | 0.862 | -0.4%  (-5.1% to 4.3%) | 0.863 |
| **Skipped Medication Doses to Save Money**^3^ | **Low Education** | 4348280 | 4.9% | 5.4% | 1.0%  (-3.0% to 5.1%) | 0.618 | 1.6%  (-4.8% to 8.0%) | 0.618 |
|  | **Higher Education** | 1723051 | 7.5% | 5.5% | -2.1%  (-8.1% to 3.9%) | 0.485 | -2.5%  (-9.5% to 4.5%) | 0.486 |
|  | **Overall** | 6071331 | 5.6% | 5.4% | 0.3%  (-3.2% to 3.7%) | 0.885 | 0.4%  (-4.5% to 5.3%) | 0.885 |
| **Took Less Medication to Save Money**^3^ | **Low Education** | 4348280 | 7.0% | 5.7% | -1.0%  (-5.7% to 3.6%) | 0.666 | -1.6%  (-8.9% to 5.7%) | 0.667 |
|  | **Higher Education** | 1723051 | 4.2% | 3.9% | -0.4%  (-5.7% to 4.9%) | 0.886 | -0.4%  (-6.6% to 5.7%) | 0.886 |
|  | **Overall** | 6071331 | 6.2% | 5.2% | -0.8%  (-4.4% to 2.9%) | 0.677 | -1.1%  (-6.3% to 4.1%) | 0.678 |
| **Financial Strain Due to Medical Bills** | | | | | | | | |
| **Get Sick or Have Accident, Worry about Paying Medical Bills** | **Low Education** | 5072274 | 56.9% | 40.6% | -14.7%  (-23.0% to -6.5%) | <0.001 | -23.0%  (-36.3% to -9.8%) | <0.001 |
|  | **Higher Education** | 2047722 | 37.0% | 28.4% | -7.5%  (-18.7% to 3.7%) | 0.190 | -8.9%  (-22.2% to 4.4%) | 0.189 |
|  | **Overall** | 7119996 | 51.4% | 37.0% | -12.9%  (-19.9% to -6.0%) | <0.001 | -18.6%  (-28.6% to -8.5%) | <0.001 |
| **Problems Paying Medical Bills** | **Low Education** | 5074787 | 22.7% | 13.9% | -6.5%  (-13.1% to 0.2%) | 0.059 | -10.1%  (-20.7% to 0.5%) | 0.062 |
|  | **Higher Education** | 2034416 | 10.2% | 5.7% | -3.9%  (-10.7% to 3.0%) | 0.265 | -4.6%  (-12.7% to 3.5%) | 0.267 |
|  | **Overall** | 7109204 | 19.2% | 11.5% | -5.8%  (-10.8% to -0.7%) | 0.025 | -8.3%  (-15.6% to -1.0%) | 0.026 |
| **Unable to Pay Medical Bills^4^** | **Low Education** | 933924 | 72.1% | 48.6% | -21.8%  (-42.2% to -1.5%) | 0.035 | -40.1%  (-77.6% to -2.7%) | 0.036 |
|  | **Higher Education** | 160324 | 18.9% | 70.0% | 47.8%  (7.6% to 88.0%) | 0.020 | 51.5%  (6.5% to 96.4%) | 0.025 |
|  | **Overall** | 1094248 | 64.3% | 51.8% | -10.3%  (-29.3% to 8.7%) | 0.287 | -17.2%  (-48.6% to 14.3%) | 0.285 |

^1^ Adults with a missing response were excluded from the analysis of the respective outcome (<1% across all outcomes)

^2^ National estimates based on survey weights for the 2019 National Health Interview Survey

^3^ Among adults taking prescription medications

^4^ Among adults reporting problems paying medical bills

**Table I. Association of Medicare Eligibility and Enrollment on Health Care Access, Prescription Medications, and Financial Strain by Education Level After Adjusting for Self-Reported Clinical Comorbidities^1^**

|  |  | **Population Size^2^** | **Adults Age 64 years** | **Adults Age 66 years** | **Effect of Medicare Eligibility** | **P-Value** | **Effect of Medicare Enrollment** | **P-Value** |
| --- | --- | --- | --- | --- | --- | --- | --- | --- |
| **Health Care Access** | | | | | | | | |
| **Recent Physician Visit** | **Low Education** | 5189342 | 92.9% | 91.8% | -0.2%  (-4.7% to 4.3%) | 0.926 | -0.3%  (-6.9% to 6.3%) | 0.926 |
|  | **Higher Education** | 2098667 | 91.5% | 93.0% | 1.6%  (-4.3% to 7.5%) | 0.593 | 2.0%  (-5.3% to 9.3%) | 0.593 |
|  | **Overall** | 7288009 | 92.5% | 92.2% | 0.3%  (-3.3% to 3.9%) | 0.870 | 0.4%  (-4.6% to 5.5%) | 0.870 |
| **Delayed Medical Care Due to Cost** | **Low Education** | 5187451 | 9.8% | 5.2% | -3.7%  (-7.8% to 0.4%) | 0.079 | -5.4%  (-11.6% to 0.7%) | 0.080 |
|  | **Higher Education** | 2098667 | 4.5% | 0.9% | -3.6%  (-6.8% to -0.5%) | 0.024 | -4.5%  (-8.4% to -0.6%) | 0.025 |
|  | **Overall** | 7286117 | 8.3% | 3.9% | -3.8%  (-6.9% to -0.7%) | 0.017 | -5.3%  (-9.7% to -1.0%) | 0.017 |
| **Did Not Seek Medical Care Due to Cost** | **Low Education** | 5187451 | 10.1% | 3.9% | -5.0%  (-8.8% to -1.1%) | 0.012 | -7.3%  (-13.1% to -1.5%) | 0.013 |
|  | **Higher Education** | 2098667 | 3.3% | 0.3% | -3.1%  (-5.7% to -0.5%) | 0.019 | -3.8%  (-7.1% to -0.6%) | 0.020 |
|  | **Overall** | 7286117 | 8.2% | 2.8% | -4.6%  (-7.4% to -1.7%) | 0.002 | -6.4%  (-10.4% to -2.4%) | 0.002 |
| **Prescription Medications** | | | | | | | | |
| **Delayed Filling Prescription to Save Money**^3^ | **Low Education** | 4444058 | 7.3% | 5.6% | -1.0%  (-5.2% to 3.2%) | 0.655 | -1.4%  (-7.7% to 4.9%) | 0.656 |
|  | **Higher Education** | 1758646 | 7.1% | 4.8% | -1.4%  (-7.0% to 4.3%) | 0.638 | -1.7%  (-8.6% to 5.3%) | 0.638 |
|  | **Overall** | 6202705 | 7.2% | 5.4% | -1.2%  (-4.7% to 2.2%) | 0.483 | -1.7%  (-6.6% to 3.1%) | 0.483 |
| **Needed Prescription Medication but Did Not Get it Due to Cost** | **Low Education** | 5183600 | 8.4% | 6.9% | -0.1%  (-4.2% to 3.9%) | 0.943 | -0.2%  (-6.2% to 5.8%) | 0.943 |
|  | **Higher Education** | 2098667 | 5.9% | 5.2% | -0.5%  (-5.5% to 4.5%) | 0.847 | -0.6%  (-6.8% to 5.6%) | 0.847 |
|  | **Overall** | 7282267 | 7.7% | 6.4% | -0.3%  (-3.7% to 3.0%) | 0.837 | -0.5%  (-5.1% to 4.1%) | 0.837 |
| **Skipped Medication Doses to Save Money**^3^ | **Low Education** | 4444058 | 5.8% | 5.2% | -0.4%  (-4.3% to 3.5%) | 0.841 | -0.6%  (-6.5% to 5.3%) | 0.841 |
|  | **Higher Education** | 1758646 | 7.3% | 5.3% | -1.3%  (-7.0% to 4.4%) | 0.659 | -1.6%  (-8.6% to 5.4%) | 0.658 |
|  | **Overall** | 6202705 | 6.2% | 5.2% | -0.7%  (-4.2% to 2.7%) | 0.682 | -1.0%  (-5.8% to 3.8%) | 0.682 |
| **Took Less Medication to Save Money**^3^ | **Low Education** | 4444058 | 7.9% | 5.5% | -2.2%  (-6.7% to 2.2%) | 0.318 | -3.3%  (-10.0% to 3.3%) | 0.320 |
|  | **Higher Education** | 1758646 | 4.1% | 3.8% | -0.3%  (-4.9% to 4.3%) | 0.905 | -0.3%  (-6.0% to 5.3%) | 0.905 |
|  | **Overall** | 6202705 | 6.8% | 5.0% | -1.6%  (-5.0% to 1.9%) | 0.370 | -2.2%  (-7.1% to 2.7%) | 0.371 |
| **Financial Strain Due to Medical Bills** | | | | | | | | |
| **Get Sick or Have Accident, Worry about Paying Medical Bills** | **Low Education** | 5186829 | 56.5% | 40.9% | -14.7%  (-22.9% to -6.6%) | <0.001 | -21.7%  (-34.1% to -9.4%) | <0.001 |
|  | **Higher Education** | 2096172 | 37.8% | 26.7% | -11.2%  (-21.9% to -0.4%) | 0.042 | -13.8%  (-27.3% to -0.4%) | 0.044 |
|  | **Overall** | 7283001 | 51.3% | 36.7% | -13.9%  (-20.8% to -7.1%) | <0.001 | -19.5%  (-29.2% to -9.7%) | <0.001 |
| **Problems Paying Medical Bills** | **Low Education** | 5189342 | 22.9% | 14.1% | -7.3%  (-13.8% to -0.7%) | 0.029 | -10.7%  (-20.4% to -1.0%) | 0.030 |
|  | **Higher Education** | 2084917 | 10.0% | 5.5% | -4.6%  (-10.8% to 1.6%) | 0.144 | -5.6%  (-13.2% to 2.0%) | 0.146 |
|  | **Overall** | 7274259 | 19.3% | 11.6% | -6.4%  (-11.4% to -1.5%) | 0.011 | -9.0%  (-15.9% to -2.1%) | 0.011 |
| **Unable to Pay Medical Bills^4^** | **Low Education** | 962757 | 73.3% | 47.5% | -23.5%  (-42.9% to -4.2%) | 0.017 | -44.4%  (-82.2% to -6.7%) | 0.021 |
|  | **Higher Education** | 160324 | 18.9% | 70.0% | 53.8%  (11.0% to 96.5%) | 0.014 | 57.8%  (11.2% to 104.3%) | 0.015 |
|  | **Overall** | 1123081 | 65.5% | 50.7% | -12.8%  (-30.6% to 5.0%) | 0.159 | -21.7%  (-52.0% to 8.6%) | 0.160 |

^1^ Adults with a missing response were excluded from the analysis of the respective outcome (<1% across all outcomes)

^2^ National estimates based on survey weights for the 2019 National Health Interview Survey

^3^ Among adults taking prescription medications

^4^ Among adults reporting problems paying medical bills

**Table J. Association of Medicare Eligibility and Enrollment on Health Care Access, Prescription Medications, and Financial Strain by Education Level After Adjusting for Self-Reported Comorbidities, Employment, other Baseline Characteristics**^1^

|  |  | **Population Size**^2^ | **Adults Age 64 years** | **Adults Age 66 years** | **Effect of Medicare Eligibility** | **P-Value** | **Effect of Medicare Enrollment** | **P-Value** |
| --- | --- | --- | --- | --- | --- | --- | --- | --- |
| **Health Care Access** | | | | | | | | |
| **Recent Physician Visit** | **Low Education** | 5029329 | 93.2% | 91.5% | -1.1%  (-5.9% to 3.6%) | 0.642 | -1.7%  (-8.9% to 5.5%) | 0.643 |
|  | **Higher Education** | 2029583 | 91.3% | 92.7% | 2.4%  (-3.4% to 8.1%) | 0.417 | 2.8%  (-4.0% to 9.7%) | 0.417 |
|  | **Overall** | 7058912 | 92.7% | 91.9% | -0.2%  (-4.1% to 3.7%) | 0.929 | -0.3%  (-5.7% to 5.2%) | 0.929 |
| **Delayed Medical Care Due to Cost** | **Low Education** | 5029329 | 9.4% | 5.3% | -2.6%  (-6.7% to 1.5%) | 0.216 | -3.9%  (-10.1% to 2.3%) | 0.217 |
|  | **Higher Education** | 2029583 | 4.6% | 0.9% | -3.0%  (-5.8% to -0.1%) | 0.042 | -3.5%  (-6.9% to -0.1%) | 0.043 |
|  | **Overall** | 7058912 | 8.0% | 4.0% | -2.8%  (-5.9% to 0.3%) | 0.079 | -3.9%  (-8.2% to 0.5%) | 0.080 |
| **Did Not Seek Medical Care Due to Cost** | **Low Education** | 5029329 | 9.4% | 3.9% | -3.7%  (-7.4% to 0.0%) | 0.051 | -5.5%  (-11.2% to 0.1%) | 0.053 |
|  | **Higher Education** | 2029583 | 3.4% | 0.3% | -2.7%  (-5.1% to -0.4%) | 0.024 | -3.2%  (-6.0% to -0.4%) | 0.024 |
|  | **Overall** | 7058912 | 7.7% | 2.8% | -3.4%  (-6.1% to -0.7%) | 0.013 | -4.8%  (-8.6% to -1.0%) | 0.014 |
| **Prescription Medications** | | | | | | | | |
| **Delayed Filling Prescription to Save Money**^3^ | **Low Education** | 4307372 | 6.5% | 5.9% | 0.5%  (-3.7% to 4.6%) | 0.823 | 0.7%  (-5.6% to 7.0%) | 0.823 |
|  | **Higher Education** | 1704468 | 7.3% | 5.0% | -0.4%  (-4.9% to 4.2%) | 0.879 | -0.4%  (-5.8% to 4.9%) | 0.879 |
|  | **Overall** | 6011839 | 6.7% | 5.6% | 0.2%  (-3.2% to 3.5%) | 0.921 | 0.2%  (-4.5% to 4.9%) | 0.921 |
| **Needed Prescription Medication but Did Not Get it Due to Cost** | **Low Education** | 5029329 | 7.8% | 6.7% | 0.6%  (-3.3% to 4.5%) | 0.758 | 0.9%  (-5.0% to 6.9%) | 0.758 |
|  | **Higher Education** | 2029583 | 6.1% | 5.4% | 2.5%  (-2.3% to 7.4%) | 0.309 | 3.0%  (-2.8% to 8.8%) | 0.307 |
|  | **Overall** | 7058912 | 7.3% | 6.3% | 1.0%  (-2.2% to 4.1%) | 0.551 | 1.3%  (-3.1% to 5.8%) | 0.551 |
| **Skipped Medication Doses to Save Money**^3^ | **Low Education** | 4307372 | 4.9% | 5.4% | 0.7%  (-3.0% to 4.4%) | 0.725 | 1.0%  (-4.6% to 6.6%) | 0.725 |
|  | **Higher Education** | 1704468 | 7.5% | 5.6% | -0.9%  (-5.6% to 3.9%) | 0.714 | -1.0%  (-6.6% to 4.5%) | 0.714 |
|  | **Overall** | 6011839 | 5.7% | 5.5% | 0.3%  (-2.9% to 3.5%) | 0.847 | 0.4%  (-4.1% to 4.9%) | 0.847 |
| **Took Less Medication to Save Money**^3^ | **Low Education** | 4307372 | 7.1% | 5.7% | -1.3%  (-5.5% to 2.8%) | 0.534 | -2.0%  (-8.3% to 4.3%) | 0.535 |
|  | **Higher Education** | 1704468 | 4.2% | 4.0% | 0.1%  (-4.4% to 4.5%) | 0.978 | 0.1%  (-5.1% to 5.3%) | 0.978 |
|  | **Overall** | 6011839 | 6.3% | 5.2% | -0.8%  (-4.1% to 2.6%) | 0.653 | -1.1%  (-5.8% to 3.6%) | 0.653 |
| **Financial Strain Due to Medical Bills** | | | | | | | | |
| **Get Sick or Have Accident, Worry about Paying Medical Bills** | **Low Education** | 5026815 | 56.3% | 40.5% | -12.8%  (-20.7% to -5.0%) | 0.001 | -19.4%  (-31.5% to -7.2%) | 0.002 |
|  | **Higher Education** | 2027088 | 37.0% | 27.4% | -6.9%  (-17.6% to 3.7%) | 0.200 | -8.3%  (-20.9% to 4.4%) | 0.199 |
|  | **Overall** | 7053904 | 50.9% | 36.6% | -12.0%  (-18.6% to -5.4%) | <0.001 | -16.8%  (-26.2% to -7.5%) | <0.001 |
| **Problems Paying Medical Bills** | **Low Education** | 5029329 | 22.0% | 14.0% | -3.4%  (-9.5% to 2.7%) | 0.275 | -5.1%  (-14.4% to 4.1%) | 0.277 |
|  | **Higher Education** | 2015833 | 10.2% | 5.7% | -3.7%  (-10.1% to 2.7%) | 0.256 | -4.4%  (-12.0% to 3.2%) | 0.256 |
|  | **Overall** | 7045162 | 18.8% | 11.5% | -3.4%  (-8.1% to 1.2%) | 0.143 | -4.8%  (-11.3% to 1.7%) | 0.145 |
| **Unable to Pay Medical Bills^4^** | **Low Education** | 910336 | 71.9% | 48.6% | -14.4%  (-31.5% to 2.7%) | 0.098 | -28.2%  (-61.9% to 5.6%) | 0.101 |
|  | **Higher Education** | 160324 | 18.9% | 70.0% | 68.9%  (40.3% to 97.5%) | <0.001 | 74.5%  (42.1% to 107.0%) | <0.001 |
|  | **Overall** | 1070660 | 63.9% | 51.8% | -3.9%  (-19.4% to 11.6%) | 0.619 | -6.7%  (-33.2% to 19.8%) | 0.620 |

^1^ Adults with a missing response were excluded from the analysis of the respective outcome (<1% across all outcomes)

^2^ National estimates based on survey weights for the 2019 National Health Interview Survey

^3^ Among adults taking prescription medications

^4^ Among adults reporting problems paying medical bills

**Table K. Association of Medicare Eligibility Health Care Access, Prescription Medications, and Financial Strain by Income Level – Continuity-Based Regression Discontinuity Analysis with 3-Year Bandwidth^1,2^**

|  |  | **Effect of Medicare Eligibility (95% CI)** | **Effect of Medicare Eligibility**  **(95% “honest” CI)** |
| --- | --- | --- | --- |
| **Health Care Access** | | | |
| **Recent Physician Visit** | **Low-income** | -10.5%  (-19.7% to -1.2%) | -10.5%  (-19.9% to -1.1%) |
|  | **Higher-income** | 3.1%  (-3.8% to 10.1%) | 3.1%  (-4.0% to 10.2%) |
|  | **Overall** | -2.6%  (-8.2% to 3.1%) | -2.6%  (-8.4% to 3.3%) |
| **Delayed Medical Care Due to Cost** | **Low-income** | -10.3%  (-20.5% to -0.1%) | -10.3%  (-21% to 0.3%) |
|  | **Higher-income** | -2.2%  (-6.9% to 2.5%) | -2.2%  (-7.7% to 3.3%) |
|  | **Overall** | -5.9%  (-11.2% to -0.6%) | -5.9%  (-12.0% to 0.2%) |
| **Did Not Seek Medical Care Due to Cost** | **Low-income** | -13.1%  (-23.2% to -3.1%) | -13.1%  (-23.4% to -2.9%) |
|  | **Higher-income** | -4.9%  (-8.7% to -1.1%) | -4.9%  (-9.2% to -0.5%) |
|  | **Overall** | -8.7%  (-13.6% to -3.8%) | -8.7%  (-14.0% to -3.4%) |
| **Prescription Medications** | | | |
| **Delayed Filling Prescription to Save Money**^3^ | **Low-income** | -4.2%  (-14.5% to 6.1%) | -4.2%  (-14.5% to 6.2%) |
|  | **Higher-income** | -3.2%  (-9.0% to 2.7%) | -3.2%  (-9.1% to 2.8%) |
|  | **Overall** | -3.8%  (-9.2% to 1.5%) | -3.8%  (-9.3% to 1.6%) |
| **Needed Prescription Medication but Did Not Get it Due to Cost** | **Low-income** | -5.6%  (-16.1% to 4.8%) | -5.6%  (-16.1% to 4.9%) |
|  | **Higher-income** | -1.7%  (-6.9% to 3.5%) | -1.7%  (-7.0% to 3.7%) |
|  | **Overall** | -3.6%  (-9.0% to 1.8%) | -3.6%  (-9.1% to 1.9%) |
| **Skipped Medication Doses to Save Money**^3^ | **Low-income** | -0.5%  (-10.6% to 9.5%) | -0.5%  (-10.6% to 9.5%) |
|  | **Higher-income** | -2.8%  (-8.5% to 2.8%) | -2.8%  (-8.6% to 3.0%) |
|  | **Overall** | -2.1%  (-7.3% to 3.0%) | -2.1%  (-7.4% to 3.1%) |
| **Took Less Medication to Save Money**^3^ | **Low-income** | -7.8%  (-18.6% to 3.1%) | -7.8%  (-18.7% to 3.1%) |
|  | **Higher-income** | -1.5%  (-5.6% to 2.6%) | -1.5%  (-5.7% to 2.6%) |
|  | **Overall** | -4.4%  (-9.5% to 0.7%) | -4.4%  (-9.6% to 0.8%) |
| **Financial Strain Due to Medical Bills** | | | |
| **Get Sick or Have Accident, Worry about Paying Medical Bills** | **Low-income** | -16.0%  (-31.9% to -0.1%) | -16.0%  (-32.1% to 0.2%) |
|  | **Higher-income** | -17.3%  (-30.0% to -4.7%) | -17.3%  (-30.3% to -4.4%) |
|  | **Overall** | -17.6%  (-28.3% to -7.0%) | -17.6%  (-28.7% to -6.6%) |
| **Problems Paying Medical Bills** | **Low-income** | -15.2%  (-29.9% to -0.4%) | -15.2%  (-30% to -0.3%) |
|  | **Higher-income** | -2.9%  (-9.6% to 3.8%) | -2.9%  (-9.8% to 4.0%) |
|  | **Overall** | -8.8%  (-16.7% to -0.9%) | -8.8%  (-16.9% to -0.7%) |
| **Unable to Pay Medical Bills^4^** | **Low-income** | -36.8%  (-69.8% to -3.8%) | -36.8%  (-73.4% to -0.2%) |
|  | **Higher-income** | 16.4%  (-37.4% to 70.1%) | 16.4%  (-39.7% to 72.4%) |
|  | **Overall** | -24.6%  (-53.5% to 4.3%) | -24.6%  (-57.5% to 8.3%) |

^1^ Adults with a missing response were excluded from the analysis of the respective outcome (<1% across all outcomes)

^2^ National estimates based on survey weights for the 2019 National Health Interview Survey

^3^ Among adults taking prescription medications

^4^ Among adults reporting problems paying medical bills

**Table K. Association of Medicare Eligibility on Health Care Access, Prescription Medications, and Financial Strain by Income Level – Continuity-Based Regression Discontinuity Analysis with 8-year Bandwidth**^1,2^

|  |  | **Effect of Medicare Eligibility (95% CI)** | **Effect of Medicare Eligibility**  **(95% “honest” CI)** |
| --- | --- | --- | --- |
| **Health Care Access** | | | |
| **Recent Physician Visit** | **Low-income** | -1.3%  (-6.6% to 3.9%) | -1.3%  (-9.3% to 6.7%) |
|  | **Higher-income** | 1.0%  (-2.3% to 4.3%) | 1.0%  (-5.2% to 7.3%) |
|  | **Overall** | 0.1%  (-2.8% to 3.0%) | 0.1%  (-5.9% to 6.1%) |
| **Delayed Medical Care Due to Cost** | **Low-income** | -9.6%  (-15.3% to -3.8%) | -9.6%  (-21.1% to 2.0%) |
|  | **Higher-income** | -2.1%  (-4.7% to 0.4%) | -2.1%  (-11.0% to 6.7%) |
|  | **Overall** | -5.3%  (-8.2% to -2.5%) | -5.3%  (-14.5% to 3.8%) |
| **Did Not Seek Medical Care Due to Cost** | **Low-income** | -9.8%  (-15.6% to -4.1%) | -9.8%  (-19.4% to -0.2%) |
|  | **Higher-income** | -1.4%  (-3.5% to 0.8%) | -1.4%  (-7.9% to 5.2%) |
|  | **Overall** | -5.0%  (-7.8% to -2.2%) | -5.0%  (-12.1% to 2.1%) |
| **Prescription Medications** | | | |
| **Delayed Filling Prescription to Save Money**^3^ | **Low-income** | -2.5%  (-7.9% to 2.9%) | -2.5%  (-9.6% to 4.7%) |
|  | **Higher-income** | -0.2%  (-3.2% to 2.8%) | -0.2%  (-5.3% to 5.0%) |
|  | **Overall** | -1.1%  (-4.0% to 1.7%) | -1.1%  (-6.2% to 3.9%) |
| **Needed Prescription Medication but Did Not Get it Due to Cost** | **Low-income** | -4.1%  (-9.6% to 1.3%) | -4.1%  (-11.4% to 3.1%) |
|  | **Higher-income** | 0.7%  (-1.8% to 3.2%) | 0.7%  (-4.1% to 5.5%) |
|  | **Overall** | -1.4%  (-4.2% to 1.3%) | -1.4%  (-6.5% to 3.6%) |
| **Skipped Medication Doses to Save Money**^3^ | **Low-income** | 1.7%  (-3.4% to 6.9%) | 1.7%  (-5.3% to 8.8%) |
|  | **Higher-income** | -0.9%  (-3.7% to 1.8%) | -0.9%  (-6.0% to 4.1%) |
|  | **Overall** | 0.2%  (-2.4% to 2.8%) | 0.2%  (-4.8% to 5.1%) |
| **Took Less Medication to Save Money**^3^ | **Low-income** | 0.5%  (-5.1% to 6.0%) | 0.5%  (-6.1% to 7.0%) |
|  | **Higher-income** | -0.8%  (-3.0% to 1.4%) | -0.8%  (-4.5% to 2.9%) |
|  | **Overall** | -0.3%  (-3.0% to 2.5%) | -0.3%  (-4.4% to 3.9%) |
| **Financial Strain Due to Medical Bills** | | | |
| **Get Sick or Have Accident, Worry about Paying Medical Bills** | **Low-income** | -11.9%  (-20.3% to -3.5%) | -11.9%  (-24.9% to 1.1%) |
|  | **Higher-income** | -13.8%  (-20.5% to -7.1%) | -13.8%  (-25.4% to -2.3%) |
|  | **Overall** | -13.5%  (-18.8% to -8.2%) | -13.5%  (-23.9% to -3.1%) |
| **Problems Paying Medical Bills** | **Low-income** | -7.3%  (-14.5% to -0.2%) | -7.3%  (-16.9% to 2.2%) |
|  | **Higher-income** | -2.5%  (-5.8% to 0.8%) | -2.5%  (-8.7% to 3.8%) |
|  | **Overall** | -4.7%  (-8.5% to -1.0%) | -4.7%  (-11.4% to 1.9%) |
| **Unable to Pay Medical Bills^4^** | **Low-income** | -9.5%  (-28.4% to 9.4%) | -9.5%  (-59.6% to 40.6%) |
|  | **Higher-income** | 20.1%  (-9.4% to 49.6%) | 20.1%  (-38.7% to 78.9%) |
|  | **Overall** | -1.2%  (-17.3% to 14.9%) | -1.2%  (-48.9% to 46.6%) |

^1^ Adults with a missing response were excluded from the analysis of the respective outcome (<1% across all outcomes)

^2^ National estimates based on survey weights for the 2019 National Health Interview Survey

^3^ Among adults taking prescription medications

^4^ Among adults reporting problems paying medical bills

**Fig A. Measures of Access and Affordability by Income-Level, Recent Physician Visit, Age 57 to 73 Years**

The proportion of US adults who reported a recent physician visit

**Fig B. Measures of Access and Affordability by Income-Level, Delayed Filling Prescription to Save Money, Age 57 to 73 Years**

The proportion of US adults who reported delaying filling a prescription to save money

^*^ Among adults taking prescription medications

**Fig C. Measures of Access and Affordability by Income-Level, Needed Prescription Medication but Did Not Get it Due to Cost, Age 57 to 73 Years**

The proportion of US adults who reported needing a prescription but not getting due to costs

**Fig D. Measures of Access and Affordability by Income-Level, Skipped Medication Doses to Save Money, Age 57 to 73 Years**

The proportion of US adults who reported skipping medication doses to save money

^*^ Among adults taking prescription medications

**Fig E.** **Measures of Access and Affordability by Income-Level, Took Less Medication to Save Money, Age 57 to 73 Years**

The proportion of US adults who reported taking less medication to save money

^*^ Among adults taking prescription medications

**Fig F. Measures of Access and Affordability by Income-Level, Unable to Pay Medical Bills, Age 57 to 73 Years**

The proportion of US adults who reported being unable to pay medical bills.

^*^ Among adults reporting problems paying medical bills
